# Supplementary material for: Conserved structural features of RNA export pores spanning the double membrane of arterivirus and coronavirus replication organelles
Source: J Virol. 2026 Aug 26;100(9):e00859-26. doi: 10.1128/jvi.00859-26 (PMC13595946; doi:10.1128/jvi.00859-26)
Supplement: Supplementary file 1 [file jvi.00859-26-s0001.pdf]

Supplementary Table 1:
Arteriviruses* used for analysis of nsp2 and nsp3 sequences, including abbreviations used and GenBank accession numbers

Virus name	Abbreviation	Accession number
Zambian malbrouck virus 1	ZMV	NC_048209.1
Free state vervet virus	FSVV	NC_029992.1
simian hemmorrhagic encaphalitis virus	SHEV	NC_038293.1
Kibale red colobus virus 1	KRCV-1	NC_033553.1
Kibale red colobus virus 2	KRCV-2	NC_034455.1
Mikumi yellow baboon virus 1	MYBV-1	NC_025112.1
Kafue kinda chacma baboon virus	KKCBV	NC_029053.1
simian hemorrhagic fever virus 1	SGFV	NC_003092.2
Kibale red-tailed guenon	KRTGV	NC_038292.1
de Brazza's monkey arterivirus	DeMAC	NC_026509.1
Pebjah virus	PBJV	NC_027124.1
Rat arterivirus 1 isolate Ningxia2015	RtClonAV	NC_032987.1
Rat arterivirus 1 isolate Jilin2014	RtMrufAV	NC_028963.1
Rodent arterivirus isolate RtEi-Arterivirus/SX2014	RtEiAV	NC_048210.1
Rodent arterivirus isolate RtMc-Arterivirus/Tibet2014	RtMcAV	NC_040535.1
Porcine reproductive and respiratory syndrome virus 2	PRRSV-2	NC_038291.1
Lelystad virus	PRRSV-1	NC_043487.1
Rodent arterivirus isolate RtClan-Arterivirus/GZ2015	RtClanAV	NC_075972.1
Lactate dehydrogenase-elevating virus	LDV	NC_001639.1
African pouched rat	APRAV	NC_026439.1
Oliver's shrew virus	OSV	NC_035127.1
Equine arteritis virus	EAV	NC_002532.2
Wobbly possum disease virus	WPDV	NC_026811.2

*all 23 known arteriviruses as described in the MSL41[ICTV]

Supplementary Table 2:
Amino acid sequences used for AlphaFold predictions

protein (domain) used for AlphaFold prediction	amino acid sequence
EAV nsp2	GYNPPGDGACGYRCLAFMNGATVVSAGCSSDLWCDDELAYRVFQLSPTFTVTIPGGRVCPNAKYAMICDKQHWRVKRAKGVGLCLDESCFR<br>GICNCQRMSGPPPAPVSAAVLDHILEAATFGNVRVVTPEGQPRPVPAPRVRPSANSSGDVKDPAPVPPVPKPRTKLATPNPTQAPIPAPRTR<br>LQGASTQEPLASAGVASDSAPKWRVAKTVYSSAERFRTELVQRARSVGDVLVQALPLKTPAVQRYTMTLKMMRSRFSWHCDVWYPLAVIACL<br>LPIWPSLALLLSFAIGLIPSVGNNVVLTALLVSSANYVASMDHQCEGAACLALLEEEHYYRAVRWRPITGALSLVLNLLGQVGYVARSTFDAAYVP<br>CTVFDLCSFAILYLCRNRCWRCFGRCVRVGPATHVLGSTGQRVSKLALIDLCDHFSKPTIDVVGMATGWSGCYTGTAAMERQCASTVDPHSF<br>DQKKAGATVYLTPPVNSGSALQCLNVMWKRPIGSTVLGEQTGAVVTAVKSISFSPPCCVSTTLPTRPGVTVVDHALYNRLTASGVDPALLRVG<br>QGDFLKLNPGFRLIG
EAV nsp3	GWIYGICYFVLVVVSTFTCLPIKCGIGTRDPFCRRVFSVPVTKTQEHCHAGMCASAEGISLDSLGLTQLQSYWIAAVTSGLVILLVCHRLAISALDL<br>LTLASPLVLLVFPWASVGLLLACSLAGAAVKIQLLATLFVNLFFPQATLVTMGYWACVAALAVYSLMGLRVKVNVPMCVTPAHFLLLARSAGQSR<br>EQMLRVSAAAPTNSLLGVARDCYVTGTTRLYIPKEGGMVFE
EAV-Y-domain	CRNRCWRCFGRCVRVGPATHVLGSTGQRVSKLALIDLCDHFSKPTIDVVGMATGWSGCYTGTAAMERQCASTVDPHSFDQKKAGATVYLTP<br>PVNSGSALQCLNVMWKRPIGSTVLGEQT
PRRSV2 nsp2	EGNCGWHCISAIANRMVNSKFETTLPERVRPPDDWATDEDLVNAIQILRLPAALDRNGACTSAKYVLKLEGEHWTVTVTPGMSPSLLPLECVQ<br>GCCGHKGGLGSPDAVEVSGFDPACLDRLAEVMHLPSSAIPAALAEMSGDSDRSASPVTTVWTVSQFFARHSGGNHPDQVRLGKIISLCQVIE<br>DCCCSQNKTNRVTPEEVAAKIDLYLRGATNLEECLARLEKARPPRVIDTSFDWDVVLPGVEAATQTIKLPQVNQCRALVPVVTQKSLDNNSVP<br>LTAFSLANYYYRAQGDEVRHRERLTAVLSKLEKVVREEYGLMPTEPGPRPTLPRGLDELKDQMEEDLLKLANAQTTSDMMAWAVEQVDLKTW<br>VKNYPRWTPPPPPPKVQPRKTKPVKSLPERKPVPAPRRKVGSDCGSPVSLGGDVPNSWEDLAVSSPFDLPTPPEPATPSSELVIVSSPQCIFR<br>PATPLSEPAPIPAPRGTVSRPVTPLSEPIPVPAPRRKFQQVKRLSSAAAIPPYQDEPLDLSASSQTEYEASPPAPPQSGGVLGVEGHEAEETLSEI<br>SDMSGNIKPASVSSSSSLSSVRITRPKYSAQAIIDSGGPCSGHLQEVKETCLSVMREACDATKLDDPATQEWLSRMWDRVDMLTWRNTSVYQ<br>AICTLDGRLKFLPKMILETPPPYPCEFVMMPHTPAPSVGAESDLTIGSVATEDVPRILEKIENVGEMANQGPLAFSEDKPVDDQLVNDPRISSR<br>RPDESTSAPSAGTGGAGSFTDLPPSDGADADGGGPFRTVKRKAERLFDQLSRQVFDLVSHLPVFFSRLFYPGGGYSPGDWGFAAFTLLCLFLC<br>YSYPAFGIAPLLGVFSGSSRRVRMGVFGCWLAFAVGLFKPVSDPVGAACEFDSPECRNILHSFELLKPWDPVRSLVVGPVGLGLAILGRLLGGA<br>RCIWHFLLRLGIVADCILAGAYVLSQGRCKKCWGSCIRTAPNEVAFNVFPFTRATRSSLIDLCDRFCAPKGMDPIFLATGWRGCWAGRSPIEQP<br>SEKPIAFAQLDEKKITARTVVAQPYDPNQAVKCLRVLQSGGAMVAKAVPKVVKVSAVPFRAPFFPTGVKVDPDCRVVVDPDTFTAALRSGYST<br>TNLVLGVGDFAQLNGLKIRQISKPSG
PRRSV2 nsp3	GGPHLMAALHVACSMALHMLAGIYVTAVGSCGTGTNDPWCANPFAVPGYGPGSLCTSRLCISQHGLTLPLTALVAGFGIQEIALVVLIFVSIGG<br>MAHRLSCKADMLCVLLAIASYVWVPLTWLLCVFPCWLRCFSLHPLTILWLVFFLISVNMPSGILAMVLLVSLWLLGRYTNVAGLVTPYDIHHYTS<br>GPRGVAALATAPDGTYLAAVRRAALTGRTMLFTPSQLGSLLE
PRRSV2 Y-domain	QGRCKKCWGSCIRTAPNEVAFNVFPFTRATRSSLIDLCDRFCAPKGMDPIFLATGWRGCWAGRSPIEQPSEKPIAFAQLDEKKITARTVVAQP<br>YDPNQAVKCLRVLQSGGAMVA

Supplementary Table 3:
Primers used for mutagenesis of pEAN551 and pcDNA-EAVHAnsp23GFP

Mutant	Polarity	Sequence	Genome position
EAV-R659K	fw	GACGCTGTGTGAAGGTGGGCCCTGCCACGCATGTTTTGGGCTCC	2188-2231
EAV-R659K	rev	TGCGTGGCAGGGCCCACCTTCACACAGCGTCCGAAGCATCTCC	2218-2176
EAV-R659A	fw	GACGCTGTGTGGCAGTGGGCCCTGCCACGCATGTTTTGGGCTCC	2188-2231
EAV-R659A	rev	TGCGTGGCAGGGCCCACTGCCACACAGCGTCCGAAGCATCTCC	2218-2176
EAV-R673K	fw	CCACCGGGCAAAAGGTTTCGAAACTGGCGCTCATTGATTTGTGTG	2230-2274
EAV-R673K	rev	AGCGCCAGTTTCGAAACCTTTTGCCCGGTGGAGCCCAAAACATGC	2260-2216
EAV-R673A	fw	CCACCGGGCAAGCAGTTTCGAAACTGGCGCTCATTGATTTGTGTG	2230-2274
EAV-R673A	rev	AGCGCCAGTTTCGAAACTGCTTGCCCGGTGGAGCCCAAAACATGC	2260-2216
EAV-R920K	fw	TGGTCTGCCACAAGCTTGCCATCAGCGCCTTGGACTTGTTG	2971-3011
EAV-R920K	rev	GCGCTGATGGCAAGCTTGTGGCAGACCAACAAGATCACTAATCC	2998-2955
EAV-R920A	fw	TGGTCTGCCACGCGCTGGCCATCAGCGCCTTGGAC	2971-3005
EAV-R920A	rev	CTGATGGCCAGCGCGTGGCAGACCAACAAGATCACTAATCC	2995-2955
EAV-R1043K	fw	CTTGGAGTGGCCAAGGATTGTTATGTCACAGGCACAACTCGGC	3339-3381
EAV-R1043K	rev	ACATAACAATCCTTGGCCACTCCAAGCAGTGAATTGGTGGGGG	3364-3322

Supplementary Table 4:
Primers used for RT-PCR amplification of the EAV nsp2-nsp3 coding sequence

Name	Polarity	Sequence	Genome position
EAV nsp2 CTD	fw	CCATGACTCTGAAGATGATGCG	1750-1771
EAV nsp2 CTD	rev	GCCACATTTGATAGGTAAGCAGG	2792-2770
EAV nsp3	fw	AACCGGTTGACTGCTTCAGG	2625-2644
EAV nsp3	rev	TGTGCCGTAGCTGCTACC	3482-3465

Supplementary Figure 1

>ref|NC_048209.1|:Zambian_malbrouck_virus_1_nsp2/612-1219
DGSCGIHIFEQIMAHMRGRPPNYLKFDRRYDDWATTLDVADLATAARLPVGGSNCVHMRYRVTLFDNHFRVE
VNEDGSTGFSRQCSSFACDPSVPVDILNCDSVDITGWYEHHNGASSASDAMKLACQLCNYLLEEPGAPPPRR
ATVLKFVGAALKSEPAQLFGQKKKPTPKLVATPASVVTSEAPASDPPATSAQSASSNADTTPLLSAQEQPAA
DDPAPSPKLVQEIEGTVARPKSRFRPPDWQALRRYITTTCAHYCHVGRAPDFSRFLPYLLSLWNSSRKTLTG
VDLAAAAFLLLIGLLLAVLPTVVFIVPALILAIRARPHWVSYAGPLGMGVAYVGSVLLGPPPNACIVDDGRC
DTALRALSTRFSAGHGHSYTLGPVSFVHVLFKYFVHLEADLHLLSSVLCMLDLLLCVLWLFRRRYCWRCFAP
CIRTAPAEIPLRAVPLTRVSSTTLLDICDNFSRPPVDIIKMATGYKGCYCGQLPCMTEGIKPVSYSKLDTKK
VSPRTVIPVPTTPNEAVKAIAVLNARGVMARSAHTVERVDKLPCKNPFFPYDLNLKRIVVVDAETMDLFSDL
GVDVSHLVVGDGDFFKVMGVARPPANVAIKGG
>>ref|NC_029992.1|:Free_state_vervet_virus_nsp2/616-1218
DGSCGLHIFEQIMAHMNSREPNYRGCQRAYENWATTLDVADLATAARIPAGGMNCVHMRYRITLHDSHFRVE
VNKSGSLGFSRQCAGFACDPSVPVDICNSDSVDLTGWYEHHNGTVSATEYCRLGVQLCNFLLKEPGAPPPRR
QNVLKAVSAAINNKPTHLGIIKPNPAALTGKAESKAKTANPPPPKEKDSDKVPLLSAEASSPTESDTTPEVK
VEQAPDPVLNDLATPKPRFWPPDWTKFRRDVTTACANYCHVARAPNFSRFLPYLLSLWNTTRTTLSGVDLAV
ACVLLFVGLLLSVLPTVVFVVPAVLLCYWTRPHWISYVGPLGMAALFVGTVLLGPSPNACAVDDGYCDSALL
VLAQRFSAGRPVRYSLGSLSFVFILYEYFVYVETDLSLLSTLLCLCDLVLCTLWVVRRCHCWRCFAPCIRCA
PGEIPLRTVPISRVSNSTLLDICDNFSSPPVDIIRMATGYKGCYRGSLPCVTEGAKPISYSKLDMKKLSPRT
VVPFPTTPAEAVKAIHVLNARGVMARSPHTVERVDKLPCKNPFFPYDVNSRRIVAVDAETYSLFKDLGLDLS
HLVIGEGDFFKCMGVVRPPAQNAVSGG
>ref|NC_038293.1|:simian_hemorrhagic_encephalitis_virus-_nsp2/632-1235
DGSCGIHVFEQINAAMNNRPPAYIGVGRAYED-ASTLDVADLATAARLPVGGESCVHMRY-IMLRDSHFAVQ
ENPSGSLGFSRQCASFACDSSVPVEIVNSDYVDLRGWYEHHNGVTNLTSYFKLGIKLCNQLLKEPGAVPPRR
ADVLKAVSASINQKPIRSLEPAVKPSAPTPAVQPTAPAHDGKKEHNEKTPLFSAEASTPATDQATPAACDTT
PAAPPAADIGLSDVGKPRARLRVDWTSFRRKATQACAHYCHVARGRDFSRCLPYLLSFWNTTRASLHGVRLA
VASLLLLVGLLLAVMPTMVFVVPALLLIYWTRPHWVSYAGPAGMGAIYLMSLLLGPSPNACSTDSGHCDTAL
HALAARFANRRAGRHSLGPTSTLFVLYEYFVYMEADLSLISSLLCVFDVVLCCAWLLRRRYCWRCFAPCIRT
APSEVVLRTVPLSRVSLSTLLDICDNCARPPVDVIKMATGYSGCYCGNLPTVTAGTKPISYSKLDLKKLSPR
TVIPIPSTPAEAVKAIHVLNARGVMTPLVHLVEKVDKLPCKNPFFPYDLNNKSVVAVDPDTYSLFSELGLDL
SHLVIGEGDFFKAMGVKRPASSAVVRGG
>ref|NC_033553.1|:Kibale_red_colobus_virus_1_nsp2/609-1199
DGGCGVHVIHQLLCHIKGLTPSYPKMQWAYAQWLDNDQMGQVICSLKLPVGQGSCVHCQYVVNIVDQHWELR
YHKDRAFVHSPLCAHGICHTVVGPIECVENPRYVDISGLLEFSDKFSSGTEFTKLASALGENGHCKRVSPRR
KWYLELLSRKTATDVPLVESSGPSAPPLIEGPAVLPISTSSNANNSNSSASVPAQDSACPEQQDKDGSSTAP
ADDSGTVPVRLKDRVKHWAFDKYVDFNRQVSHIKPHLLHFLHGSNRAVHPGTYCSSLLLMCICMLLCLHAQF
VGIALLAVPLYLCHARQSIRVLSLFAVFLYVAALFLSDEHHLCAVDDHQCLQFLHDLQRRFSTSPPRFLTPG
PITAGAVFVNYFWGAVVLGYFLYYLGFLIDIGLVGLVAGHNRICWRCFRPCIRTAPEELVLLTVPASRIHRA
TVLDVCDNYSNPPVDIVKQATGYAGCYRGQLPSVASVCCNVPAAKVDVKKVSAKTVCSIPTSPSEAVKALQV
LNARGQLGYENHAVEAVAVLPCANPFFPYDTSSSKIVTVCAYTYNLFTELGVDTSLLTIGDGDFFKAMGVEK
PKSFCLAALKKRVGG
>ref|NC_034455.1|:Kibale_red_colobus_virus_2_nsp2/590-1186
DGSCGYHIICQIISHMLGREPAYVKYSRDRAQWMDNFNLFKIVQSIRLPVGFNNCPHMKYVIRLTNDHWEVY
HNQSVREAYSQGCASGYCLEVKCPINAIENSNWLDISGLYNEHPNFDSPDEFGKVVQSLAAHFIDQGDEKVP
KIRAKTLLDIMKRGRQLKGEENQPLLPQPAEEEKPKPTPRLRSQASDKSTSSSQSANATITSEGPAVTSGEE
IKTVLDDLLNSSQPRGKLATLKEDIQKGLTQVGDQVSRFQPHLLAFLCSPSSRVSMPVLVFSHFLLLLACFF
ALAPSIWAVPITAGAWVLAGRHRSVRAITALVLCFLCFSTVFPEAESVCESDGPDCRDRLTALANRFRGAVV
RYVGAGPGTCILLFARSLYYFESAASMLYALLFIFDLALLCCILSHNRICLRCYGCCIRKAPEEVVLKTIPL
SRISRSTLLDICDSFSRPPVDIILQATGWRGCYTGCFNPVVDSASPIPVSRVDPKKITSNTICTLPVCPAEA
VKAIHVLHNRGQLAFDRTAKVEKVETLPCKNPFFPYDVMSRKIVVVDSATFELFTQLGLDTSMLVLGQGDFF
EAMGVARPTKLAAAALRVRGG
>ref|NC_025112.1|:Mikumbi_yellow_baboon_virus_1_nsp2/565-1189
DGGCGVHCLYAISEHRATKQWPSIKPMVDWAYESWADDTQMGDVACALALPVGFEGCTHQPYIIRLVDAHFV
VDYYPARVASYSPHCAHRTCMSLVGSTMVLEATPGRFSDFHKLLPRYKKAENFLAHLQGLAGTKAVCHHSRP
TPRAATLCKVIDTPPCKPANVSASKLISIDKIATVDAIKPQPAKIAPVPPPRKARKVSADASPNTEADSQPV
TPQLGGVNNETSDLPVPPDTTNPPQATATSPQEPTKSSPITWHDRLLQGTLKNAAAKATNVVSRLHPQLLAY
LDMRDVKAGNTTSYYISSGLWALTLLLLSISPFLGLALGILAFVCCPNSRTSRVSSLLYPLLLFCKLVLSEQ
SLVCESDDSGCRDFLLSVSLRYASAPPRFVTPGPFTAGFAVLRNFVIVTTLVQYAHFLLLVFDVCILLLCMC
HRSFCLKCYGRCIRLAPEEIQGRCVPSSRLSRVSLVDICDVYKAPPCDIIKMATGYAGCYTGSLSCVKACSS

ALPVAHIDPKKVSNTTSCTFPTCASEAVKALAVLSARGTLSLGKPPRVVKVEKFPCKNPFFPYDLSLTTPVV
VDCATYELFAELGLNVSHLVIGEGDIFQVMNVKRPTFYEKARLKLTKGG
>ref|NC_029053.1|:Kafue_kinda_chacma_baboon_virus_nsp2/566-1189
DGGCGIHALAAVAHHLRHKSWPKIEPMVNWGYQQWADSEIMGELACDLGLPLGFESCTHQPYIIALVDNHFV
VRHYPSRTPTHSPTCQHGCCHTLVGTATVPTYGQAGDFNVFHKLLPRFKDAAEFIRMARALLCQNRVCHKPI
PPVRKTALVKLMATTPSTAAKVPPSEVRSILKPQRPTPDPRTKSTKAAPAPPPAPASPPETPPDPDLSDVEL
GQNSAAGVPDECAPPLASETPEVAAPPQSKIRRTVADAHYFMTTSLNNAFIAAHDKATTQVSRVMPHLLSAI
SFSGGQRPRGMVLFGSLFLWFLAYLICLLGNPCGIPLGFAAFYLAPKLKSSIFFSILLPVVLLYQRLLSPSY
MVCEAATPECDDYLRSLVDVFSNPPPKFVTPGPCTLALAIVRSYTRIPSGVYFLHILALTCDLLIFCLLMYY
NSICHKCFSFCIRKAPEEIQLCTVPSSRVSKHTLLDICNNYQRPPVDVVQMATGYRGCYTGVLNSTINSNSS
IPCTKIDPKKVTNSTVCTMPSCASEAARAICVLSARGHLSRGGLAKVVKVEKLPCKNPFFPYDLASSTPVVV
DNYTFELLSDLGVNVSLLTVGEGDFFAAMGVARPSFFEKAQLKLLRGG
>ref|NC_003092.2|:simian_hemorrhagic_fever_virus-nsp2/639-1236
DGGCGVHAFAAIQYHINTGHWPEQKPVVNWAYEAWTTNEDIGHMICSTETPAALEPCLHARYVVRLDSDHWV
VDHYPNRPMCFVEACAHGWCSSLLSEPTGEEGEHLVDCSALYDCLGKFRNGTEFADTVLGLSKTAHCCNKRV
PTPRKQAIMSLLNRPNCVPCIAPPSQVRTVDPSQPAAPLPPVPRPRKRKAAAQQVSKVPSEQDPSLAHDPPE
KPDSVRPPKLGYLDRAWNNMLARTHKLHNLQQRVFGLYPQLLSMLLPSGARPSTPRLLGCYFSMAVAMFFLF
LGSPLFILCAVLAGVIAPSARYPKILCCCLVVVYICTLFADAISSVCDNDDADCRAFLSDLGDRYSTNQPVY
ITPGPATFFLAVSRNFFVVSVALFPLHLLLLMVDVLLVIGVLCMDGYCFRCFSRCVRKAPEEVSLLTIPQSR
VSRRFLLDICDFYSAPPVDIIRLATGLNGCFRGDYSPIGSSTSVITADKIDVKKVSCRTVCSFPSCPSEAVK
VLHVLSVRGQMCAHNEQKVEKVDALPCKNPLFPYDLSSKKIVPVDSGTYEILSSIGCDMSHLVIGDGDFFKV
MGVPRPSPFTVMRLRACRVVGG
>ref|NC_038292.1|:Kibale_red-tailed_guenon_virus_nsp2/571-1188
DGGCGVHCVAAIQHHVINKCWPAQSPKVNWGIQQWTDSDDLGEFLITSGTPAAIAPCNHARYVIRLVDNHFV
VDHYPQRPALLSPVCCRGFCISVVGSVPGVEKEDCTFDITGSYSLLNRFNSAKEFFNVTTDVIKNRSSDVQL
LGIVDRQGNPSASVPHSYVTAVTPNSEADFIKFSKPAPAPRKRKAATPLAAVVTEDQPQSSTAPPEQPKESS
TAQPAKAEKVNDPSPPPSDEKDAHGIKSRYKTARTWCARACRDAISALNDPSGRVFGLLPHILAFFHCQTQP
VSLWRLLVAYLSVALALVFTYHRSILATIFMVIPLLVCPKSARTRLISVVCGIIFLLNLFLGSERALCESDD
DQCLRAIDHLRLRFSTTAPVYVTPGLLTVGFALLRNFIYTGAASVYIHCLLLLADIVLILALMFLNGICLRC
FGRCIRTAPHEVHLCTIPASRVSRATLLDICDTFSAPPIDIIRMATGYAGCYQGCINPTGAAATIECARVDP
KKVTPSTCASFPSCASEAVKAIHVLSARGTIGPFNNAKVEKVEKLPFKNPLFPYDVDNKKVVVVDPTTYTLF
SELGCDVSHLVLGSGDFFKAMNTPRPDAFTILKLKATRRLGG
>ref|NC_026509.1|:DeBrazzas_monkey_arterivirus_nsp2/591-1210
DGACGVHCLAAIQHHIANGVWPTQQPVVDWAYEQWLDSDSLGDMIVATGTPAAIAPCDHARYVIALVDSHWI
VRFYPDRELFRASACQRGFCISAVGPVGGVTVKQPKSVAVGLYSLLGRFQSGEEFGRVLVALSGGKWRGVAA
SISDSELLRIVDSAAATPAKVPASYVTTLPVKDEKDMAQNAIPTTQHKDTAPSVAEVKVPDAAPSVETQPPQ
TPATASNAAAPLAAENTNNNAAPGTGPPPKRKLTWRERGNNYLARLHNVIADPAGRVFHLYPQLLALLAPRQ
NRYPLSRLVCAYSLFALALVCLSFGSWFCFLFGAAALGCIWSSRHARALFGILVVCFVLRLFADESSSLCEH
PDDRCHEYLDSVRGRLSASFRTFITPGLLTVFLAFFRGLYPITSALFVLHYFLLLLDLVFILALLFVRRICL
RCWGRCIRTAPEEVPLLTIPSSRVTRAFLLDIANTFSSPQVDVIRMATGYAGCYDGCVCPTGTSANIAVGKV
DVKKVTHKTSCSVPTCPTEAVKALHVLASRGTIAPLNNNKVKKVDALPCRNPLFPFDIKNKVITCVDSDTYS
LLSELGCDLSHLIIGTGDFFQEMGVPRPDYFTVLRLKAARIMGG
>ref|NC_027124.1|:Pebjah_virus_nsp2/596-1189
DGGCGLHAVQAIACHYRTGKWPEQSAKVCWDEKKWTDSDQLAELIGALHLPAGLDMGSGCSHCRYVISLESD
HWVVFYYDNRPAAFSSCCAAGACASAVGTLAGMESPTWYDPSGVYDLLPCIESPEEYCNVVCGLVPRASLTK
PRPVLRQRVLASLAVRPANEPAKVPCSYVREIHSKSDLNDCIPPKPNEAPCPASTDAIEKAQAAQEIARDTV
APDKPVGRAPFLLRATSYLQQHAAAASSRVFQYKPHLLAALSTSSGSARSPMVYVGFGLFSLGFLLVGLSPL
ASILLGCCGLLFCFTSRTGITMFASLVCVSILNLVLRSSSPLCENRDDDCVAYLHQLRDRYDDPPSVYLTPG
PFTAFFAFIRWLRFVSLRPLLVHCILLLLDSLLLLLILLSHKVCRHCWRRCIRLAPEEIVLPTIPSSRTSRA
VLLDIADAFAPPPVDVIRLATGYSGCFRGVLPAVGVGSGVISCDAIDTKKVRANTLCSMPSTPAEAVKAIHV
LSARGQLAYQSNCKVEKVEKLPCRNPIFEYDVNSRNIVTVDAKTFELLRDLGCDTSHLIIGSGDFFEVMGVK
RPDRLGVALMQARRISGG
>ref|NC_032987.1|:RtClon_arterivirus_nsp2/431-1533
DGNCGWHCLSAVANRMISNVFESSLPTRFRDSHDWASDDDLVSVISAAGLPVGLNRCGYCCDAKYVLHLTDD
HWTVTCNPGVAPKQLPLACIHGVCQHVGRQVGGSPPPVDKIHPGFNSMLESLMSLPSSAIALGMQELLAVPA
DTESARSVRRSDVSMMLPNNIDLQLPAVPGEQVSALDLSRSDVGTPTDSVRETWTVSQALARLDTNDAKKFC
DIRDLLVGLFAASGVTQQEVDDAINASIASCSNLSECCAVLEKSLCRTAVTKSKCDLTAYFPEYRPANPCRA
VVPVKPVKTVTKETGWTPHGLDVRPQQVSVPLSAETVMNFTYPGCADAHYAITGVCRRISLGIREKYGFGKR

HPTDPWYPDTPQDLTDLIDKAEEDFTQMAGAQLSLENMIWFCQEFDVERHVASYTWTPCDAPPEVPPDSQVQ
RRRHTVDAPTGSVAVVAAPAVEVPDSWEDLCDGGASQPLPLDFTLGTLSSVSAEPVQCEPLDLSTSGSVKFG
PPASQCAGITSADLGPLAPPECGSVLDLSGSSTTVFDLTQRNESPTPSSPASSASSAVLPVERPPKSAQALI
DAQGDMCRTLTEIKFRARNMCLAACDPTRLTDPATEAWVNCMWDRLDLLTWKNKSKYQAAFQLGDLSMLPGM
ILETPPPYPCPVMLPLGSPPRSATPESDVTLRTLSEHPPCPPGACTEKDASAPALADSEDSPPPAPAGSDPV
PPPGDSQAPSRSTFCGGIFASAASTAAGLCDGFSRQVFAVASHLPAFFARAFHSGGGYTSGDWCFAAFVLCC
LLLCYSYPPVGCAPLLGVFSGSGRRVRIGVFGVWLAFAIILFRPTPDPIAAACDSDSPDCRRVLLDFERQQP
WDPVRSLVVGPLGLGTAILGRLLGGARYFWFVLLRLCFLGDLLLAGAYIVSQGRCKRCWGRCVRMAPSEIPL
NVFPFTRATRTSLVDLCNRYCAPKGIDPIFVASGWRGCYSGDSPIEQPSSTPISYVNLDEKKISSRTVVSPP
YDPSQAIKCLKVLQAGGAMAACRVPAVVKLTQVPFLAPFLPKLPVNPDARIVVDSETFTCALRSGYDTSNFI
LGEGNFAELNGLKIQQLQRPKGG
>ref|NC_028963.1|:RtMruf_arterivirus_nsp2/433-1634
DGNCGWHCLSAVCNRMLVGAFQSSLTAAARPSQDWATDEDLVQAAQRLGLPVGLYRNGACPDAKYVLSLKND
HWIVTCKPGMAPKRLPPECAHGVCQHIGSQIGGAAEPCIIRNDTAALLQHAIHLPSSAIPDALVHMMMPNHN
KLKVLTPADVARRSDVAASAAAVPASLSGAPTTSVQPAVPGETCADSAPVDLTRRSDVATPVAAVPAGPPSV
ADDVLDLSRSDVGSVSSEVVEHWTVSQILSRTGLPDCDKFASVVDLAHALFTMTGATPEEINAAIDATIVKC
ASVEECCAAVQKCLKDVAITKQGFALGNYFPDYQPKNTCTAIVPVADKVKIKEFVPGSTLREAMAGIGTYVE
KEVPVTVDRVRHGLCPGGEQADWAMGDICRKIRDEMRNTYGYGWDPVYRWYPDAPADLLDIIEKAETDFLAM
CDAQASLAGMKWFADNFDVAAHVANYSWVPQIVTKQPKVVKAKQPDRRRTPTPVAPAPPAEPEVPDSWEDLA
TEDQPVDLSPAPAMVAGGNPQFAPLTFGAKEASWGSECESIDQPLDLSVKGSIKFGPPSSQNSVCLTDMESV
VLEDPAPALLADVPLDLSCSSVTVFDAAASNSSPAQSPPESVFGDEEKQVIRPPKSAQALIDAGGDLAKHLI
IIKKRAMDMCRQACDPNYVDHPSCVIWCESMWNRLDMLTWHNKSKYQASYQLATFEQFLPRMIQETPPPHPC
PIMWEPASPPRSKTPSVDLTIRSGVTTPRSRASSLRQEEQKIDAAGVADVAPTSEDEEKLAPEPQLAQPATL
AGKWRNIFRRDPAAQAEKKSEEVTPPPETTKPPDEGGTPPGNDTTWSRIGAKCGEFTDKLCGKVFEITSHLP
AFFARAFHSGGGYTAGDWCFAAFVLCCLLLCYSYSAFGCAPLLGVFSGSPRRTRIGVFGVWMAFAALLFRPL
QDPVGSACDSDSPECRRLLLAFEQLQPWKPVRGLVVGPFGLTASLLGKLLGGPRNFWAILLRLMFMADVIAA
GAYIVGQGRCKKCWGRCIRTAPQEVAMNVFPFTRATRQSLVDICNRFCSPKLIDPIFVATGWRGCYIGQSPI
EQPSSKPFTYQNLDEKKISATTIVTPPYDPTQAIKCLKVLQAGGAIAATKVPEVTKIDSVPFLAPFLPKLPV
NPDVKIVVDTETFTCAIRSGYDTSALILGEGDFAKENGMKIQQLQRPSGG
>ref|NC_048210.1|:RtEi-Arterivirus_nsp2/350-1520
DGSCGWHCLSAVANRMMGKEFKSTLPVVHRPSSDWATDADLVDAILIGHLPVGLNRNGACVDAKYVLTLDGS
HWTVACRPGQTPKMLPEACVHGVCEHVGSQIGGNPLPVEGFNPAFNDVLQRVIYLPSVAIADALATMLVNPA
EMAADGQGGRRDVTEECVDSAFPSDDSELRCDHSGDVLSLVAAFHAVPGTGSDVVEDVAEEAVSPVTAPVDL
TVKSPSKRCETSTPEERGEGRRDVTGGGNPATCPVAAPVGPLSTLENEPLEDTSIQWTTSQILNRPGLAADS
LFLDLVTVFEAAFAPAGASQAEVHAAIDATINGAKSYSECLQRLLPVAQGLKVTKDKVSLEAYFPKYVPVNS
CRAIVPVPAVEYVKDRVLGKGKWEVVGKPVLLTLENVMNCSFPGSYETYSAICSAKQRIQNALRDKYGYGKV
NPADKWYPDMPDSLVDVVLQAEDDFLKVVGAQGTLERMTWMAQTFPATEWADRYVWIADEQPVAVQQPPKKN
RRAAQPAVAAEAAPPAQEVVEEVPESWEELADPVDLSSRSCGHLPPLLFGAPTVASNEQPDDQPLDLSAAGS
IKFGPPPSQDEVEVCDFESVLSDPVVAQPLDLSGSSVTEFRAPVAKASSAPSTTSTVAKRPAKSAQALIDAK
GPLSGHLQQIKDNVRKICIAATDPNFETHPQTQAWLDAMWNRVDLLTWKNTSKYQAAYQLAPVSSYLPKMIM
ETPPPYPCPIVLTPPDPPKSETESVDLTIRSRVSSKPATPTPPPQMEEVPLCEDPEDLQQSSKPPDPPGEES
KAQADQPNQPKESLATKAGTWAGKMFDKMCGQVFNVTSHLPAFLAAAFHPGGGYTPADWCFAGFTLCCLLLC
YSYPAFGCAPLVGVFSGSARRVRMGCFGVWLAFAVLLFKPTDDPVGSACDSDTPECRQLLLAFEQRQPWEPV
RGLVVGPIGLTASILGKLLGGARSVWWILLRLCFVADVVCAGAYVIGQGRCKKCWGRCIRTAPSEVAFNVFP
FTRGTRQSLVDLCDRFCKPKGMDPIQLACGWSGCWSGESPIEQPSSKPISYQALDEKKISASTVVAQPYDPN
QAIKCLKVLQAGGAMVAIKVPTVTKVNTVPFLAPFLPKCPVNPDVKIVVDPDTFTTAIRCGYDVSSFVLGEG
DFAAANGLKVQQMQRPSGG
>ref|NC_040535.1|:RtMc-Arterivirus_nsp2/428-1521
EGNCGWHCLAAVINRMKNDVFKTFLAERVRNSDDWATDGQLADAILIMGLPVGFDRGRVCPNAKYVMKLQGE
HWTVSVRPGMTPKSLPLVCVQGCCSSVPHEAAPLTFSPDCDRALEDAMMLPSSCVPAGLSDWLNRASPVLEK
WTVAQIYARYECCKSDKSYGSFLSYHQLLQNTMCGDPKKGRVTEEELNDAIDQLIDGSGDIKECLARLTKVR
APSLFASSVKLNDFFPDYVPAASHTAQAPPVVEQQNVCMALVPVKPTGSIVREAAPAERLGCKDRLKNVSVP
LSVQSVMDFTYPNCHDVHYRITEVLRRAEWCLLARYGQVYQYAPTSFRDMIDECKSELLAIADRQCTLEMMA
NVLEDIDITAFTAKYKWTPENEPAPRLVETRQQRRRPQTLRSTASEPAKKKVEEVPECWESLADSEPCDLSA
VSKGGQRAKFANVSTEENDVEIPVVEQPLCLKAGTTLGPASAAPPATLRPQHEVSAPTDASSDWTTTSASSA
LQDEPLDLSGSSQTEWMIVSQPTFVPKTRPNTPVADEVFDDVPLDLSSKPGSSASSVVSEPVVMRPKNSAQA
IIDANGPLSSHLAAIKRNVRKVCQQACDPNSLNEPETKDWLDHMWERVDMLTWHNTSRYQASYQLASMDYLP
KMILETPPPKPCEVVFAPADTPSQSLQSESDITLNDLGSMSGICSVTPSQLHEKLAQMEQAPPGPSKDLAA

PKASEDPPPPGKIKGRFEKIVNSACDQVFALCTHLPSFFAQVFRPGGGYTSGDWAFAAFTLCCLLIAYSYPA
FGCTPLVGVFSGSARRVRMGVFGCWLAFAVVLFQVDSEPVGAACSSDTPECRDLLLAFEQRQLWNPVRSLIL
GPWGAVAAILGRILGGPRYFWHCLLRFGFLADVLFYCFYIVSQGRCKRCWGQCIRTAPSEVPFNVFPFTRAT
RQSLVTLCDRFSRPKGMDPIHLATGWHGCWTGQSPIEQPCDKPITYANLDEKKVSAQTVVAQPYDPNQAVKC
LRVLQSGGAMVAEAVPKVVKVQTVPFLAPFFPKVRVDADTKIVVDPPTFSAALRSGYSTSALIIGEGDFAKI
NGVKVGCYVAPSGG
>ref|NC_038291.1|:Porcine_reproductive_and_respiratory_syndrome_virus_2_n
sp2/434-1579
EGNCGWHCISAIANRMVNSKFETTLPERVRPPDDWATDEDLVNAIQILRLPAALDRNGACTSAKYVLKLEGE
HWTVTVTPGMSPSLLPLECVQGCCGHKGGLGSPDAVEVSGFDPACLDRLAEVMHLPSSAIPAALAEMSGDSD
RSASPVTTVWTVSQFFARHSGGNHPDQVRLGKIISLCQVIEDCCCSQNKTNRVTPEEVAAKIDLYLRGATNL
EECLARLEKARPPRVIDTSFDWDVVLPGVEAATQTIKLPQVNQCRALVPVVTQKSLDNNSVPLTAFSLANYY
YRAQGDEVRHRERLTAVLSKLEKVVREEYGLMPTEPGPRPTLPRGLDELKDQMEEDLLKLANAQTTSDMMAW
AVEQVDLKTWVKNYPRWTPPPPPPKVQPRKTKPVKSLPERKPVPAPRRKVGSDCGSPVSLGGDVPNSWEDLA
VSSPFDLPTPPEPATPSSELVIVSSPQCIFRPATPLSEPAPIPAPRGTVSRPVTPLSEPIPVPAPRRKFQQV
KRLSSAAAIPPYQDEPLDLSASSQTEYEASPPAPPQSGGVLGVEGHEAEETLSEISDMSGNIKPASVSSSSS
LSSVRITRPKYSAQAIIDSGGPCSGHLQEVKETCLSVMREACDATKLDDPATQEWLSRMWDRVDMLTWRNTS
VYQAICTLDGRLKFLPKMILETPPPYPCEFVMMPHTPAPSVGAESDLTIGSVATEDVPRILEKIENVGEMAN
QGPLAFSEDKPVDDQLVNDPRISSRRPDESTSAPSAGTGGAGSFTDLPPSDGADADGGGPFRTVKRKAERLF
DQLSRQVFDLVSHLPVFFSRLFYPGGGYSPGDWGFAAFTLLCLFLCYSYPAFGIAPLLGVFSGSSRRVRMGV
FGCWLAFAVGLFKPVSDPVGAACEFDSPECRNILHSFELLKPWDPVRSLVVGPVGLGLAILGRLLGGARCIW
HFLLRLGIVADCILAGAYVLSQGRCKKCWGSCIRTAPNEVAFNVFPFTRATRSSLIDLCDRFCAPKGMDPIF
LATGWRGCWAGRSPIEQPSEKPIAFAQLDEKKITARTVVAQPYDPNQAVKCLRVLQSGGAMVAKAVPKVVKV
SAVPFRAPFFPTGVKVDPDCRVVVDPDTFTAALRSGYSTTNLVLGVGDFAQLNGLKIRQISKPSGG
>ref|NC_043487.1|:Porcine_reproductive_and_respiratory_syndrome_virus_1_n
sp2/426-1463
DGSCGWHVLAAIMNRMINGDFTSPLTQYNRPEDDWASDYDLVQAIQCLRLPATVVRNRACPNAKYLIKLNGV
HWEVEVRSGMAPRSLSRECVVGVCSEGCVAPPYPADGLPKRALEALASAYRLPSDCVSSGIADFLANPPPQE
FWTLDKMLTSPSPERSGFSSLYKLLLEVVPQKCGATEGAFIYAVERMLKDCPSSKQAMALLAKIKVPSSKAP
SVSLDECFPTDVLADFEPASQERPQSSGAAVVLCSPDAKEFEEAAPEEVQESGHKAVHSALLAEGPNNEQVQ
VVAGEQLKLGGCGLAVGNAHEGALVSAGLINLVGGNLSPSDPMKENMLNSREDEPLDLSQPAPASTTTLVRE
QTPDNPGSDAGALPVTVREFVPTGPILCHVEHCGTESGDSSSPLDLSDAQTLDQPLNLSLAAWPVRATASDP
GWVHGRREPVFVKPRNAFSDGDSALQFGELSESSSVIEFDRTKDAPVVDAPVDLTTSNEALSVVDPFEFAEL
KRPRFSAQALIDRGGPLADVHAKIKNRVYEQCLQACEPGSRATPATREWLDKMWDRVDMKTWRCTSQFQAGR
ILASLKFLPDMIQDTPPPVPRKNRASDNAGLKQLVAQWDRKLSVTPPPKPVGPVLDQIVPPPTDIQQEDVTP
SDGPPHAPDFPSRVSTGGSWKGLMLSGTRLAGSISQRLMTWVFEVFSHLPAFMLTLFSPRGSMAPGDWLFAG
VVLLALLLCRSYPILGCLPLLGVFSGSLRRVRLGVFGSWMAFAVFLFSTPSNPVGSSCDHDSPECHAELLAL
EQRQLWEPVRGLVVGPSGLLCVILGKLLGGSRYLWHVLLRLCMLADLALSLVYVVSQGRCHKCWGKCIRTAP
AEVALNVFPFSRATRVSLVSLCDRFQTPKGVDPVHLATGWRGCWRGESPIHQPHQKPIAYANLDEKKMSAQT
VVAVPYDPSQAIKCLKVLQAGGAIVDQPTPEVVRVSEIPFSAPFFPKVPVNPDCRVVVDSDTFVAAVRCGYS
TAQLVLGRGNFAKLNQTPPRNSISTKTTGG
>ref|NC_075972.1|:RtClan-arterivirus_nsp2/403-1409
DGSCGWHCISAIMNHMLGKPLKSTLPSAPRPADDWASDEDLTTMIEILRLPAGIDRGSQCPVVKYNISLEDD
HWTVHCIPAMDNRHPSPECVEGVCSKTGCTTTECGDIGLADIAALNDCIGLASCQIPSAVAHLTGRADGRND
WTVYRLRHTPGEHLKDATALTDIFRSCFSEEQDVVDQHVNSFVRGLDLDQTMAALRGVRVSHGSANDLAATF
KRTEEPEEAVNTYKPGAAVNTQCMALVPAAKSKVSYNYFGWKEEELTLEAIRSGILGRDQTNREYLESIHSQ
LATRAWVDHPYPYWFYDYLQIIDHYLDEKVRVCGSAARAIDMLRVFSEDVESLVRMYPIPTPKVVKIDKAPR
PASPKVKQAPRPRPEPHNRLVWVSSDECVAYRPMADVPDSWDDEDVPLDLSSRSSRSSSSCTLAPQPIAAVV
APPPTPVCEFAQAEQVTPPTICAAEIVASLIAQTTPKKLTADEWSEQPTRFGAAIAHLKASLIDRLVRAHDP
SLKDHPAVQLWLVKVWQLINAQAASAEPWATQRFLNKQFGQINSWLRKVPPPEHLSKKESSWETLSVCSTSS
EQDEAAQAQPDEAAQAQLGEATQTQPGGVTQNQPTTTHQGDRAQASTSQPAADPPPDTSPTGDPGIKGRMGA
TFHKAFDALSMKVFQVFSHLPATASMLTKRHGNYTAREYGFAGFCLFCLLMAYYIRIFAIFPLAGCFMGSSR
IVKLAVFSTWMGFAVLLFAADAEESPLDCDSDPGECRRRLEYYVDQQPWRVVRGIVVGPVGLATAIFGQLLG
GSRYLWSCLLRLSVAFDLLACVVYVVMQGRCKKCWGMCVRVAPAEVVMKIFPLTRCNKSSLVDLCDRFCAPK
LVDPIKLTTGWAGCWSGAAPIELACSKPISYSNLDEKKIGPQTVVPPPLDPTQGIKCLKVLQAGGAIVDVAA
PKVVKVEEVPFLAPFFPKTKVNPESIIVVDVPTYTAALRCGYDVSQLVLGEGDFAKMNGLVASGPLPNKGG
>ref|NC_001639.1|:lactate_dehydrogenase-elevating_virus_nsp2/387-1287
DGACGLHCISAIINDIFGDALCTKLTNCSRDSSEWLSDQDMYQLVMTARLPATLGHCPSATYKLDCVNQHWT

```
VTKRKGDRALGGLSPECVRGVCGGECKFVPTYPREINLELAAKSPISALAFSLGVEPYCDCWNFTNSVLVND
SLAVETARAGEAYRSAMGIPKDDWVLLAELMTENCLTRREVLDKLQRGLRLHATSKPGSPASVSPASSIDFS
AAGLLLDGTESDKEAVVAVNNDCYTVLGFDKNSATKSEQELATGLFSELVEPMETSTSKHESRKILEAASRA
LKSAKPKRKRNKKKKTSSPTPTPPETPTREVPGAIEVVSGDEEAGACESATIVPDKAQARPPPRPKRQALKK
AEQGFILKDIIWNPTESGVKCLTIVEDVRAFLKSITPPGGALGTRARITAHIVEQFHVIRESTPELVLAHAE
HQAKNMHELLLSEKAKLILGIGEDTLKKLVSSQRSLPRSIGFGAWLSDQQKTADSCGEREFVEVPLKSGAEP
TPSKRDLGVSLGDQLSQDGAPRLSSSTACEIKERVPPIKDSGGGLGQKFMAWLNHQVFLLSSHLLAMWSVVL
GSRQKLNWADYVYTLFCLCCVLLCFHFPAIGFIPLAGCVFGSPWRVRLSVFSVWLCVAVVVFQEVLPEPGSV
CSSASAECAAALERYSGNGVHRPVNHIGVGLVGTVAGFVARVVGGPRHYWFYFLRLMVVLDLGLVFLAVALR
GRCKKCFCKCVRVAPHEVHLRVFPLTKVARPTLEAVCDMYSAPRVDPILVATGIKGCWQGKVSPHQVTDKPV
SYSNLEEKKISNKTVVPPPTDPQQAVKCLKVLQCGGSIQDVGVPEVKKVSKVPYKAPFFPNVSIDPECYIVV
DPVTYSAAMRGGYGVSHLIVGTGDFAEVNGLRFVSGG
>ref|NC_026439.1|:Forest_pouched_giant_rat_arterivirus_nsp2/407-1329
DGWCGLHCLSAVANSTHGAPLRSTYNPCTELAVAHWLDNFTLSEISQEFQLPICIEVAGVSCPSAAYRMRFN
NSHWEVARVPGIKRRRLSAVCIQGICQGNCPPQPPPSVGLKLRPADVHQAAPAIGAGVNWLAALRAAVNCDD
RVADSEGSCDEEYRGSTGLVFRRIRYDEFRPKHAVVPPFVPPGAPKPCRPPPPPPSTPGWSTCSSAASTDSV
DEFVLVSPNSPVTDNQPELEEVDLSSPITFGAPAVDELQGARVSVYNDDQVRELAELYVSSVKQGLKICLRD
SQVQLVESYLSAKASEGQVVGGEEPKCGTPCPQPEKLSKWPSETGTCSSPTPGVDSSDTVPLTQNRFEVPKQ
RPTPMPRGIRTGVGSSKPVPAPRTKLPRAAPESSRPCITPLVQASPSDGAAEQVTCGSEQPVVEEAKAAPVD
LLETAGATSKTLLERIKALRVSAALRLSHDGQRPAADAGDANKGGAHDSDCSGNARRAFLSCKPEDCDQTRP
DVHGKSSKEPTKAESVGNGEAVGEQKWLIRTHNPFAGAVRGVMRHVYNYMPAVFRVVPRLLQCYNGILHGSV
GISRAGACALFCCLAVSMVLVAVSPVFALLPLVLLCTRVRGRRLCFVISGMALARWIQLANTEAAVIQSCHT
GAPECLELRSFIAGNILDGPVSVATIGPFGVLGSFLAGVLGGDRYGWTLVLRAAFAVDLALVIIGFISQNRC
KRCLGNCIRTVKTAEIPTFTVPSTKVATAALVNYCDVVSKEYPDLVFMLTGVRGCMCGSRTLSPTSSTSYKN
LDPVKVGPRTVLPWPSNVTQATAALKSVLNGASICSRTPLKVTPAKTVPFKHETFPDLPVDPDQEIIVDLPT
YHLLSTQKCKMDKVVIGEGDFAAVNGILPGNGKIWANNVITPERAQIPGINLVALRNLG
>ref|NC_035127.1|:Olivier's_shrew_virus_1_nsp2/389-1122
DGGCGLNCLQLCANDVLGLPHSWCSFPLDSKDWLDDNDMALLAQWYELGGSVARNGKGCPKARYWFHSNNGH
WTVQVRPVKRCCLDYTCSMGICNGDCKKPAVSVTPSELSAVTCLTFQHIYYKIKAGATVSSLLSDSVVMQLA
PGPREGPPPPPPPPADCHCSASFPLDFGDIKVNPQVFHTRASQPKCLATSMESRPASPVQSGTVQELAKTGS
TTPGKSDNTSQSSTGATPPTGEPAVLPVTDCTASTNSSAPSGASKRGCCDPAAIVLSAEGEQPVDTEALRDL
LIEDQEHPSNILSNLGKYLWKGSKTPDYSRLVEDERCEVKSCPDFDPGDPPDESSRFDILKEWLKRRMVFAK
TPRSLASVPQAFMLLSRPGTVPLNTHDWIGLSLLAFACLGSMVVDWFWLCALLAALLHTPRGVLYAFVGLLG
FAASLLAGRRSLDLSACDAGLETCRPILQGFVLHRASGLDLVPTVGVVGVLVSIIARLVGGSTLVYHLLLRG
GVIVDMLLLGVGYYFSGVCTKCWRPCIRTAHRDVNLVCVPAAKVSRLTLTTICDLYCAPPVDFIKLATGYHG
CYTGTVSCTTQGKAIPYAKLDKNKIGNGTLIPLPVTPNQAKDAINVILNGGSFATSNLPPVIKVFSVPFRSV
YFPNLPVDPSKPIVVDQSIFARLSAEQRNQVILGSGDFVAVNKLMPPELDPVHGVLWAEKVLTPARTSWLSA
SQTQKLCDAGYAGG
>ref|NC_002532.2|:Equine_arteri_virus_nsp2/267-832
DGACGYRCLAFMNGATVVSAGCSSDLWCDDELAYRVFQLSPTFTVTIPGGRVCPNAKYAMICDKQHWRVKRA
KGVGLCLDESCFRGICNCQRMSGPPPAPVSAAVLDHILEAATFGNVRVVTPEGQPRPVPAPRVRPSANSSGD
VKDPAPVPPVPKPRTKLATPNPTQAPIPAPRTRLQGASTQEPLASAGVASDSAPKWRVAKTVYSSAERFRTE
LVQRARSVGDVLVQALPLKTPAVQRYTMTLKMMRSRFSWHCDVWYPLAVIACLLPIWPSLALLLSFAIGLIP
SVGNNVVLTALLVSSANYVASMDHQCEGAACLALLEEEHYYRAVRWRPITGALSLVLNLLGQVGYVARSTFD
AAYVPCTVFDLCSFAILYLCRNRCWRCFGRCVRVGPATHVLGSTGQRVSKLALIDLCDHFSKPTIDVVGMAT
GWSGCYTGTAAMERQCASTVDPHSFDQKKAGATVYLTPPVNSGSALQCLNVMWKRPIGSTVLGEQTGAVVTA
VKSISFSPPCCVSTTLPTRPGVTVVDHALYNRLTASGVDPALLRVGQGDFLKLNPGFRLIGG
>ref|NC_026811.2|:Wobbly_possum_disease_virus_nsp2/466-1096
DGWCGYSCLAKLNGFSGSTVKDSAIWVARFATLMKTELLDYIGRDLPADLWLTDDMIVKHTPYAVVTRDQMH
CANVLGVMINNRGHWKCRLPTYRVAPETACSGLGCVHVEPRIERFPTIKYDWVFQNSARIESQVSEQDLLNE
AFIGGKVITDQCLLSEITLTPRSSPPPQEPRKRKKRSKQKPVADPPAPKKLTDQVPEIKQPVASPVPAPRLH
KPQPIKPVVPPVPAPRTKKQRPVPAPRKLPDLVEKATPVAPAKARFAHVTNRTAAAVFEATVGWSNSLKAYV
AKLPRTCPAWYSLSMFLLMALPPGLGSVLSFVLGAVFLFLTVSPVPLVISVTLFSWFVLTRPHFIQCSSWDR
ECLATNGLPMPESIVVMNRGSAGLIGLVIHLFAYAGMSRRIFATLRVVSVAVDTLALYAAYVLDGVLCFKCF
HQCARTQKKLHSSEKAKNIVVNNTMILQFMDTYAPPPVDLVKLATGVNGCHCGSKSFIQWSTARPVAYSRYD
PTKSSAETVLPLPKNAEQALRVISHALEHGVVVFHMGHTGVDVKRIEAYPASISPLPDFFPDLPYTSTEATL
VVDVNLKAALSACGYPDMDKIVVGEGDWLLENEVYPIGVDQKRTCRYLSHHVSPG
```

Supplementary Figure 1: Sequences used for arterivirus-wide alignment of nsp2.

FASTA format sequences of nsp2 from the N-terminal border of the papain-like proteases until the nsp2/3 cleavage site from all 23 known arterivirus species as described in the MSL41 [ICTV]. The accession number, name, and corresponding pp1a amino acid numbers are indicated above the respective sequence.

Supplementary Figure 2

>ref|NC_048209.1|:Zambian_malbrouck_virus_1_nsp3/1220-1442
ANLDFVLIFAWVVVWCLAGNYLQVPTPCGFGTPNPFCRGTFVYPTTHTHGVCRYGFCMSNAGVSAQGLLDL
PRFGIGGFFVGVLIAGILGITWAPLDALVMSTLLVLPSGSLFTALRVVIIVLAAKFMNFRHICLGLALVAPF
DPYAAVTGLIILGCCVLSARYTGFGGLVTPYDIHAYTTNPRQAIALANAREGTYLASVRQAALTGKPAWFVP
SNFGIMVE
>ref|NC_029992.1|:Free_state_vervet_virus_nsp3/1219-1441
SHLDYIAIVAWIAFWCILGNYLQAPSPCGYGTPNPFCRGSYVHPVTANQGVCHHGMCMSDSGISRHSLFTL
PRLSINGFLLSALIAVVFFVTCVPLDGVVISTLLLLPVNPYCTVFRIVLLVASSAFISFKNLGIGLALIAPL
DPTAACAGLIILGCCYFTGRYTGFGGLITPYDIHAHTSNPRQAIAVANAKDGTYLAAVRQAALTGKPAWFIP
SNFGVMVE
>ref|NC_038293.1|:simian_hemorrhagic_encephalitis_virus-_nsp3/1236-1458
SYLEYVIVTAWIICWSLIGNFLQVPVPCGYGTPNPFCRGSFVYPTTHTHGICRYGYCMSDLGVSQQGLFPM
PTLSLGVFFYGVLIVLILCVTLVPIDGLVVSALLVLPPSSYTTLIRICVILLGSKFMTFRNLGICLAVVTPL
DPYASLTGAIILACCYLSSAFTGFGGLVTPYDIHRFSGNPRQAIALANAKEGTYLAAVRQAALTGKPAWFIP
SNFGVMVE
>ref|NC_033553.1|:Kibale_red_colobus_virus_1_nsp3/1200-1423
RKTALLAALAWVLFWVLLGAFLQSPVACGHGTNDRFCVNAYGVPVIGNHGVCAYGYCADPQGVMRQGILNV
PLSSITLPVIVFLAVVFAVFWKFHLLGYVYSLLPFFLPNLPLVVPLRVLICIAGSLRVPLHFNIIHLAATFY
MDPAGGVLTAVIAAAAWALGRFTGVGGLVTPYDIHRVVKSSRDAVALANAPHGTYLAAVRDAALSGHNKLFL
SNSIGYMLE
>ref|NC_034455.1|:Kibale_red_colobus_virus_2_nsp3/1187-1409
KLCQTAFVTAWIIFFVTLGYWLQLATPCGYGTGDPFCRSAYGYLVSYTQGVCYDGYCASSLGYSRQTLFIP
PIPGTFTIAVIICAIVAVNLLSKMPFDYFLSAVFLLLPTGTYYTLCRIAVFIFGMNHVSCFTVASWLAIGLI
LDINAFFVCAAVAVLGWIVSNTGCCGLVTPYDIHRIVKTSRDAVNVYNAPAGTYLHAVKMAALTNSNKFFIA
SNAGYVLE
>ref|NC_025112.1|:Mikumbi_yellow_baboon_virus_1_nsp3/1190-1416
GRLPVDLLLTGIVSFVSICIGAFFQRSSICGIGTSDPFCRNTFGVPLIAKQGVCDHGYCASPQGISASVASL
LTQYEYLPYITLFITVVLLAYWYVPQVFVIGIIFLNALAPTTPLTSVMRVCAFFVLSTKIDIKLLLLTVLTT
SLIDLPASIICFILFGSAWVIGKCTGVGGLVTPYDIHLCAKTPRDSIAIANAAPNTYLGAVRMAALSGTNRF
FVGSNAGIVLE
>ref|NC_029053.1|:Kafue_kinda_chacma_baboon_virus_nsp3/1190-1416
GFIRKEKIAAIVQAIFCALLGASVQSSRLCGIGTSDPFCADSFATPIYQQQGVCNYGYCASPQGISSSLVS
LIFTSDYLAYIVFALGLVFLAIHYVPNVVVVAALMLNTLTPVTPFTSFIRVLVMVCSSGYVEFRVLVFAIIS
SALLDPTASIVILILLSLTWAIGKWTGLGGLVTPYDIHLVSKTPRDAIAVANAPPNSYLGAVRMAALTNTNR
FYVGSNTGTVLE
>ref|NC_003092.2|:simian_hemorrhagic_fever_virus-nsp3/1237-1496
GRIFRTALAAAWVLFFVCAGYWVQMSTPCGIGTNDPFCKSSFGVPTYVNQGVCHGQYCASSKGVSRATSILT
VRNPAVAPYIVLAACLVYLASVYVPGIIEVSLLVLNALLPAGPAISALRTLVMIIAAPHLSMKYIAFFCCTT
AFVDFTSVVVVLTALLVGWILARYTGIGGFVTPYDIHDVVKSQRDGVAVANAPPNTYLGAVRRAALTGKPAF
FVANNTGIVLE
>ref|NC_038292.1|:Kibale_red-tailed_guenon_virus_nsp3/1189-1414
GIVPRAALAAAYVIACVCLGVYLQSPSHCGITTSDPFCKSSFGVPIIQSQGICRGDICLSPNGVSRSVFGA
LDLTPVAPYIIVLLLLVFVLWYHFPTLIEAGFTLLVALLPGSHAVNVLRVGLALAFAPFVSIKVLVFHICTT
MLLSPICAFVILVVLLVAWYVGNQTGTLGLVTPYDIHRVVKSPRDSVAIANAPPNTYFGAVRRAALTGKPVL
FMADNTGFIFE
>ref|NC_026509.1|:DeBrazzas_monkey_arterivirus_nsp3/1211-1437
GVAVRTVAAAAYIVVCVMLGSYLQLPTTCGISTRDPFCMSSFGVPVVASQGVCRGGYCASPLGISRNTPDL
LSLAPAVAPYIAILLLICFLLWQYVPTVVEAIAVLIAAAMPSTPLVDAIRVILFFLALPRLSTKVIGFYLAS
TVLISPAAACISAIGMACAWIVGAFTGTVGLVTPFDIHRLSRSSRDAVAIANAPANTFLGAVRKAALTGKPT
YFLADNTGIVLE
>ref|NC_027124.1|:Pebjah_virus_nsp3/1190-1417
GILHFRPLLALAWVLFFIFLGLWFQSSHACGVGTSDPFCKGSFGVPIVQQQDICDSGFCGSPFGVSRSLVH
YLTPDVSVAPYLVLAITVAYLLLSYVPGLLETVCLLINAVLPTSPLVTVIRVICFQVALPHLTQRGIVIFTV
TCALIDIPSLVTICILAISAWVLGKYTGVGGLVTPYDIRNVVKTARDSVAVANAPPNTYLGAVRRAALTGGC
VYYIADNTGIVLE
>ref|NC_032987.1|:RtClon_arterivirus_nsp3/1534-1763
FTYGMASLHAVVWVVAHMILGIYVTRPSQCGTGTSDPWCSDPFSVPVFGSGTLCTSNLCISPSGLTLPLAT
ALRDFGAREASIVGLVLLAVACVAHRLAVKADALAVLFSLVAYVYPLASWALACFPVLMRWLPLHPFTALWV

HFFLVACNPPAGGLALAFSFTCWILGRYTQVAGLVTPYDIHSHTSGPRGAAAILTAPEGTYLAAVRRAALTG
KTIMFCPSGVGSLLE
>ref|NC_028963.1|:RtMruf_arterivirus_nsp3/1635-1864
ATYVMAAVHVAVWMVLHMLAGIYVMQVDQCGAGTRDPWCSNPYSVPVFGSGTLCSRDLCISPSGLTLPLST
IIKDFGAREAGVIGLVIASLAVLAHKMSMKSDAVFVLASLFCHVHPLLAWVVALFPLTLKWFSAHPFTIVWV
IFFLVTCNPAAGILAGVFLVLLWILGRFTHVAGVVTPYDIHAYTNGPKGASALLTAPEGTYLAAVRRAALTG
RTVMFCPSNVGSLLE
>ref|NC_048210.1|:RtEi-Arterivirus_nsp3/1521-1749
GYVMPAIHVAVWMAVHMLLGLWITSVDSCGVGTRDPWCSSPFSVPVFGSGTLCTSNLCISPAGLTLPLASL
VKDFGAREVGIIGLVLASVACLAHKLAVKTDALLVLASILCHAHPMLAWVVCLYPLALRWCSVSPLTMLWVH
FFLVVCNPAAGILSVVLVIMFWTLGRFTHVAGLITPYDIHAYTNGPRGASALVTAPEGTYLAAVRRAALTGR
TVLYSPSNVGSLLE
>ref|NC_040535.1|:RtMc-Arterivirus_nsp3/1522-1751
APYLSALVHVAISIALHLIAGVYLTSVSSCGTGTNDPWCTSPFSVPVYGPGQLCSSKLCISDKGLTLPLIF
AASNLGWSEAGYIALVFASLLFVANRLALKADLILVLCAMACYVNPMLAWIVCLYPLALRHFTLHPLTILWV
QFFLVVCNAPAAMLAFVLLVCLWVLGKYTQVAGLVTPYDIHAYTGTPRGAASVASAPDGTFLAAVRKSALTG
RTMMFAPSTTGAILE
>ref|NC_038291.1|:Porcine_reproductive_and_respiratory_syndrome_virus_2_n
sp3/1580-1809
GPHLMAALHVACSMALHMLAGIYVTAVGSCGTGTNDPWCANPFAVPGYGPGSLCTSRLCISQHGLTLPLTA
LVAGFGIQEIALVVLIFVSIGGMAHRLSCKADMLCVLLAIASYVWVPLTWLLCVFPCWLRCFSLHPLTILWL
VFFLISVNMPSGILAMVLLVSLWLLGRYTNVAGLVTPYDIHHYTSGPRGVAALATAPDGTYLAAVRRAALTG
RTMLFTPSQLGSLLE
>ref|NC_043487.1|:Porcine_reproductive_and_respiratory_syndrome_virus_1_n
sp3/1464-1693
ASYTLAVAQVSAWTLVHFILGLWFTSPQVCGRGTADPWCSNPFSYPTYGPGVVCSSRLCVSADGVTLPLFS
AVAQLSGREVGIFILVLVSLTALAHRMALKADMLVVFSAFCAYAWPMSSWLICFFPILLKWVTLHPLTMLWV
HSFLVFCLPAAGILSLGITGLLWAIGRFTQVAGIITPYDIHQYTSGPRGAAAVATAPEGTYMAAVRRAALTG
RTLIFTPSAVGSLLE
>ref|NC_075972.1|:RtClan-arterivirus_nsp3/1410-1639
FGRVSACLHCLVWVCIHMLLGAYITAPGACGIDTPDPWCRNPFSRPVLRQGSVCSLDLCISPEGLSLPLQS
VLVTLGIKEWAAVALVITSLLALGQRYAVQADILQVMACILTYTFPSLSWVACLFPLELKWISLHPITIVWS
CAFLVFVNPPAGAVSITLLIAAWLLGRATNVCGLVTPYDIHSYTTTPRSVASLASAPEGTYLAAVRRAALSG
RSCMFVASGLGSVLE
>ref|NC_001639.1|:lactate_dehydrogenase-elevating_virus_nsp3/1288-1512
HVADFVCLGLYVMLNFLISAWLSSPVSCGRGTNDPWCKNPFSYPVVGQGVMCNSHLCISEDGLTSPMVLSY
SLIDWALMIAVIATVAIFIAKVSLLVDVICVFLCLLMYVFPPLSVIAFAFPFALCKVHLHPVTLVWVQFFLL
AVNFWAGVAVAVILISSWFLARATSSTGLVTPYDVHLVTSTPRGASSLASAPEGTYLAAVRRSALTGRCCMF
VPTNFGSVLE
>ref|NC_026439.1|:Forest_pouched_giant_rat_arterivirus_nsp3/1330-1558
YAAGAIVTAVCSVEMLVSYLLGLFAQRARVCGVNTNDPFCQSPYGVPVVLSQGYCDSGACISAAGYSVEAPA
IFVDSTLLTCCTLGFMAILLFCAEIVTLFDIFAPLLSIGALWYSPLQFIAAFTPVLLRFGGHRVVVLANGVF
LLLVSRPAALIYWAFLVVSYLQKFVASPAIHLITPHTLGEITNNPLAAMAVANAKPGSYQHTAKTAALTGRC
YLHIPSHASVLLE
>ref|NC_035127.1|:Olivier's_shrew_virus_1_nsp3/1123-1347
IASVWIFWYSVICLAAGLVLQQPSVCGVGTNDRFCRDPFARPTEAYQGYCHAGYCLSSAGLSTAGSLMPQG
LIGLVQTSLLFVLALAAGAKLLRLSMVELGLLLLPFCTAVHPTLHFVTLLGPVSLWWGVNTISYLVFLVIVS
FVSPVGLLGNLAFCLVAALLRFVPGAPVVITPWDLARFVSNPKNAASVANATPGSYVHALRTSALTGRNMIY
IPTTQGITLE
>ref|NC_002532.2|:Equine_arteri_virus_nsp3/833-1064
WIYGICYFVLVVVSTFTCLPIKCGIGTRDPFCRRVFSVPVTKTQEHCHAGMCASAEGISLDSLGLTQLQSY
WIAAVTSGLVILLVCHRLAISALDLLTLASPLVLLVFPWASVGLLLACSLAGAAVKIQLLATLFVNLFFPQA
TLVTMGYWACVAALAVYSLMGLRVKVNVPMCVTPAHFLLLARSAGQSREQMLRVSAAAPTNSLLGVARDCYV
TGTTRLYIPKEGGMVFE
>ref|NC_026811.2|:Wobbly_possum_disease_virus_nsp3/1097-1375
LWKNLSASDRATLKDLNYRPTSFGFSVSLCRVFALVQIVAGLASYFVPVFDCGIGTYDKWCEVPFNSPVKG
KHSLAVCSGATCVSPDGVFTRDPVFPVAICGLYISALAAAAIVISKLRISVGAIVTVIGSVLNYCFFPEYCY
AGPLIFLAQLFMPWTIQSLATAALSAFLWDPTATIVVIILVATFMVWSSCGGSMASVNINGPVVSPLLLMSN
GTAPSVVTPTTLACAAQSAGVPFASLVARAAVSRPGTVLKAAYDSAKDGKVRIYIPTGTGVVQE

Supplementary Figure 2: Sequences used for arterivirus-wide alignment of nsp3.

FASTA format sequences of nsp3 from all 23 known arteriviruses as described in the MSL41 [ICTV]. The accession number, name, and pp1a numbering were indicated above the respective sequence.

Supplementary Figure 3

```
ZMV/612-1219        612 DGSCGIHIFEQIMAHMRGR--PPNYLKF-DRRYDDWATTLDVAD 652
FSVV/616-1218       616 DGSCGLHIFEQIMAHMNSR--EPNYRGC-QRAYENWATTLDVAD 656
SHEV/632-1235       632 DGSCGIHVFEQINAAMNNR--PPAYIGV-GRAYED-ASTLDVAD 671
KRCV-1/609-1199     609 DGGCGVHVIHQLLCHIKGL--TPSYPKM-QWAYAQWLDNDQMGQ 649
KRCV-2/590-1186     590 DGSCGYHIICQIISHMLGR--EPAYVKY-SRDRAQWMDNFNLFK 630
MYBV-1/565-1189     565 DGGCGVHCLYAISEHRATKQWPSIKPMV-DWAYESWADDTQMGD 607
KKCBV/566-1189      566 DGGCGIHALAAVAHHLRHKSWPKIEPMV-NWGYQQWADSEIMGE 608
SGFV/639-1236       639 DGGCGVHAFAAIQYHINTGHWPEQKPVV-NWAYEAWTTNEDIGH 681
KRTGV/571-1188      571 DGGCGVHCVAAIQHHVINKCWPAQSPKV-NWGIQQWTDSDDLGE 613
DeMAV/591-1210      591 DGACGVHCLAAIQHHIANGVWPTQQPVV-DWAYEQWLDSDSLGD 633
PBJV/596-1189       596 DGGCGLHAVQAIACHYRTGKWPEQSAKV-CWDEKKWTDSDQLAE 638
RtClonAV/431-1533   431 DGNCGWHCLSAVANRMISNVFESSLPTR-FRDSHDWASDDDLVS 473
RtMrufAV/433-1634   433 DGNCGWHCLSAVCNRMLVGAFQSSLTAA-ARPSQDWATDEDLVQ 475
RtEiAV/350-1520     350 DGSCGWHCLSAVANRMMGKEFKSTLPVV-HRPSSDWATDADLVD 392
RtMcAV/428-1521     428 EGNCGWHCLAAVINRMKNDVFKTFLAER-VRNSDDWATDGQLAD 470
PRRSV-2/434-1579    434 EGNCGWHCISAIANRMVNSKFETTLPER-VRPPDDWATDEDLVN 476
PRRSV-1/426-1463    426 DGSCGWHVLAAIMNRMINGDFTSPLTQY-NRPEDDWASDYDLVQ 468
RtClanAV/403-1409   403 DGSCGWHCISAIMNHMLGKPLKSTLPSA-PRPADDWASDEDLTT 445
LDV/387-1287        387 DGACGLHCISAIINDIFGDALCTKLTNC-SRDSSEWLSDQDMYQ 429
APRAV/407-1329      407 DGWCGLHCLSAVANSTHGAPLRSTYNPCTELAVAHWLDNFTLSE 450
OSV/389-1122        389 DGGCGLNCLQLCANDVLGL--PHSWCSF-PLDSKDWLDDNDMAL 429
EAV/267-832         267 DGACGYRCLAFMNGATV--------VSA-GCSSDLWCDDELAYR 301
WPDV/466-1096       466 DGWCGYSCLAKLNGFSGS-------------------------- 483

ZMV/612-1219        653 LATAARLPVGG----SNCVHMRYRVTLFDNHFRVEVNEDGS--T 690
FSVV/616-1218       657 LATAARIPAGG----MNCVHMRYRITLHDSHFRVEVNKSGS--L 694
SHEV/632-1235       672 LATAARLPVGG----ESCVHMRY-IMLRDSHFAVQENPSGS--L 708
KRCV-1/609-1199     650 VICSLKLPVGQ----GSCVHCQYVVNIVDQHWELRYHKDRA--F 687
KRCV-2/590-1186     631 IVQSIRLPVGF----NNCPHMKYVIRLTNDHWEVYHNQSVR--E 668
MYBV-1/565-1189     608 VACALALPVGF----EGCTHQPYIIRLVDAHFVVDYYPARV--A 645
KKCBV/566-1189      609 LACDLGLPLGF----ESCTHQPYIIALVDNHFVVRHYPSRT--P 646
SGFV/639-1236       682 MICSTETPAAL----EPCLHARYVVRLDSDHWVVDHYPNRP--M 719
KRTGV/571-1188      614 FLITSGTPAAI----APCNHARYVIRLVDNHFVVDHYPQRP--A 651
DeMAV/591-1210      634 MIVATGTPAAI----APCDHARYVIALVDSHWIVRFYPDRE--L 671
PBJV/596-1189       639 LIGALHLPAGL-DMGSGCSHCRYVISLESDHWVVFYYDNRP--A 679
RtClonAV/431-1533   474 VISAAGLPVGLNRC-GYCCDAKYVLHLTDDHWTVTCNPGVA-PK 515
RtMrufAV/433-1634   476 AAQRLGLPVGLYRN-GACPDAKYVLSLKNDHWIVTCKPGMA-PK 517
RtEiAV/350-1520     393 AILIGHLPVGLNRN-GACVDAKYVLTLDGSHWTVACRPGQT-PK 434
RtMcAV/428-1521     471 AILIMGLPVGFDRG-RVCPNAKYVMKLQGEHWTVSVRPGMT-PK 512
PRRSV-2/434-1579    477 AIQILRLPAALDRN-GACTSAKYVLKLEGEHWTVTVTPGMS-PS 518
PRRSV-1/426-1463    469 AIQCLRLPATVVRN-RACPNAKYLIKLNGVHWEVEVRSGMA-PR 510
RtClanAV/403-1409   446 MIEILRLPAGIDRG-SQCPVVKYNISLEDDHWTVHCIPAMD-NR 487
LDV/387-1287        430 LVMTARLPATL----GHCPSATYKLDCVNQHWTVTKRKGDRALG 469
APRAV/407-1329      451 ISQEFQLPICIEVAGVSCPSAAYRMRFNNSHWEVARVPGIK-RR 493
OSV/389-1122        430 LAQWYELGGSVARNGKGCPKARYWFHSNNGHWTVQVRPVKR--C 471
EAV/267-832         302 VFQLSPTFTVTIPGGRVCPNAKYAMICDKQHWRVKRAKGVG--L 343
WPDV/466-1096           --------------------------------------------

ZMV/612-1219        691 GFSRQCSSFACDPSVPVD--ILNC-DS--VDIT----------- 718
FSVV/616-1218       695 GFSRQCAGFACDPSVPVD--ICNS-DS--VDLT----------- 722
SHEV/632-1235       709 GFSRQCASFACDSSVPVE--IVNS-DY--VDLR----------- 736
KRCV-1/609-1199     688 VHSPLCAHGICHTVVGPIECVENP-RY--VDIS----------- 717
KRCV-2/590-1186     669 AYSQGCASGYCLEVKCPINAIENS-NW--LDIS----------- 698
MYBV-1/565-1189     646 SYSPHCAHRTCMSLVGSTMVL-EA-TP--GRFS----------- 674
KKCBV/566-1189      647 THSPTCQHGCCHTLVGTATVPTYG-QA--GDFN----------- 676
SGFV/639-1236       720 CFVEACAHGWCSSLLSEPTGEEGE-HL--VDCS----------- 749
KRTGV/571-1188      652 LLSPVCCRGFCISVVGSVPGVEKEDCT--FDIT----------- 682
```

```
DeMAV/591-1210      672 FRASACQRGFCISAVGPVGGVTVKQPK--SVAV-----------  702
PBJV/596-1189       680 AFSSCCAAGACASAVGTLAGMESP-TW--YDPS-----------  709
RtClonAV/431-1533   516 QLPLACIHGVCQHVGRQVGGSPPPVDKIHPGFNSMLESLMSLPS  559
RtMrufAV/433-1634   518 RLPPECAHGVCQHIGSQIGGAAEPCIIR-NDTAALLQHAIHLPS  560
RtEiAV/350-1520     435 MLPEACVHGVCEHVGSQIGGNPLPVEGFNPAFNDVLQRVIYLPS  478
RtMcAV/428-1521     513 SLPLVCVQGCCSSVPH-----EAAPLTFSPDCDRALEDAMMLPS  551
PRRSV-2/434-1579    519 LLPLECVQGCCGHKGGLGSPDAVEVSGFDPACLDRLAEVMHLPS  562
PRRSV-1/426-1463    511 SLSRECVVGVCSEGCV---APPYPADGLPKRALEALASAYRLPS  551
RtClanAV/403-1409   488 HPSPECVEGVCSKTGCTT----TECGDIGLADIAALNDCIGLAS  527
LDV/387-1287        470 GLSPECVRGVCGGECKFVPTYPREINL-------EL--AAKSPI  504
APRAV/407-1329      494 RLSAVCIQGICQGNCPPQPPPSVGLKL---RPAD-----VHQAA  529
OSV/389-1122        472 CLDYTCSMGICNGDCKKPAVSVTPSELSAVTCL-----------  504
EAV/267-832         344 CLDESCFRGICNCQR-----------------------------  358
WPDV/466-1096           --------------------------------------------

ZMV/612-1219        719 -----GWYE-----------------------------------  722
FSVV/616-1218       723 -----GWYE-----------------------------------  726
SHEV/632-1235       737 -----GWYE-----------------------------------  740
KRCV-1/609-1199     718 -----GLLE-----------------------------------  721
KRCV-2/590-1186     699 -----GLYN-----------------------------------  702
MYBV-1/565-1189     675 -----DFHK-----------------------------------  678
KKCBV/566-1189      677 -----VFHK-----------------------------------  680
SGFV/639-1236       750 -----ALYD-----------------------------------  753
KRTGV/571-1188      683 -----GSYS-----------------------------------  686
DeMAV/591-1210      703 -----GLYS-----------------------------------  706
PBJV/596-1189       710 -----GVYD-----------------------------------  713
RtClonAV/431-1533   560 SAIALGMQELLAVPADT----ESA----------------RSVR  583
RtMrufAV/433-1634   561 SAIPDALVHMMMPNHNKLKVLTPA----------------DVAR  588
RtEiAV/350-1520     479 VAIADALATMLVNPAEMAADGQGGRRDVTEECVDSAFPSDDSEL  522
RtMcAV/428-1521     552 SCVPAGLSDWL----NR---------------------------  564
PRRSV-2/434-1579    563 SAIPAALAEMSGDSDRS---------------------------  579
PRRSV-1/426-1463    552 DCVSSGIADFLANPPPQ---------------------------  568
RtClanAV/403-1409   528 CQIPSAVAHLTGRADGR---------------------------  544
LDV/387-1287        505 SA----LAFSLGVEPYC-------------DCW--NFT--NSVL  527
APRAV/407-1329      530 PAIGAGVNWLAALRAAV-------------NCDDRVA---DSEG  557
OSV/389-1122        505 -----TFQH-----------------------------------  508
EAV/267-832             --------------------------------------------
WPDV/466-1096           --------------------------------------------

ZMV/612-1219            --------------------------------------------
FSVV/616-1218           --------------------------------------------
SHEV/632-1235           --------------------------------------------
KRCV-1/609-1199         --------------------------------------------
KRCV-2/590-1186         --------------------------------------------
MYBV-1/565-1189         --------------------------------------------
KKCBV/566-1189          --------------------------------------------
SGFV/639-1236           --------------------------------------------
KRTGV/571-1188          --------------------------------------------
DeMAV/591-1210          --------------------------------------------
PBJV/596-1189           --------------------------------------------
RtClonAV/431-1533   584 RSDVS-------------------------------------MM  590
RtMrufAV/433-1634   589 RSDVAASAAAVPASLSGAPTT---SVQPAVPGETCADSAPVDLT  629
RtEiAV/350-1520     523 RCDHSGDVLSLVAAFHAVPGTGSDVVEDVAEEAVSPVTAPVDLT  566
RtMcAV/428-1521         --------------------------------------------
PRRSV-2/434-1579        --------------------------------------------
PRRSV-1/426-1463        --------------------------------------------
RtClanAV/403-1409       --------------------------------------------
LDV/387-1287        528 VNDS----LA---------------VE-----------------  535
```

```
APRAV/407-1329      558 SCDE----------------------------------------  561
OSV/389-1122            --------------------------------------------
EAV/267-832             --------------------------------------------
WPDV/466-1096           --------------------------------------------

ZMV/612-1219            --------------------------------------------
FSVV/616-1218           --------------------------------------------
SHEV/632-1235           --------------------------------------------
KRCV-1/609-1199         --------------------------------------------
KRCV-2/590-1186         --------------------------------------------
MYBV-1/565-1189         --------------------------------------------
KKCBV/566-1189          --------------------------------------------
SGFV/639-1236           --------------------------------------------
KRTGV/571-1188          --------------------------------------------
DeMAV/591-1210          --------------------------------------------
PBJV/596-1189           --------------------------------------------
RtClonAV/431-1533   591 LPNNIDLQLP----------------AVPGE---QVSALDLSRS  615
RtMrufAV/433-1634   630 RRSDVATPVAAVP------------AGPPSV---ADDVLDLSRS  658
RtEiAV/350-1520     567 VKSPSKRCETSTPEERGEGRRDVTGGGNPATCPVAAPVGPLS-T  609
RtMcAV/428-1521         --------------------------------------------
PRRSV-2/434-1579        --------------------------------------------
PRRSV-1/426-1463        --------------------------------------------
RtClanAV/403-1409       --------------------------------------------
LDV/387-1287        536 ----------------------------------TARAGEAYRS  545
APRAV/407-1329      562 ----------------------------------------EYRG  565
OSV/389-1122            --------------------------------------------
EAV/267-832             --------------------------------------------
WPDV/466-1096           --------------------------------------------

ZMV/612-1219        723 -----------------HHNGASSA--------SDAMK------  735
FSVV/616-1218       727 -----------------HHNGTVSA--------TEYCR------  739
SHEV/632-1235       741 -----------------HHNGVTNL--------TSYFK------  753
KRCV-1/609-1199     722 -----------------FSDKFSSG--------TEFTK------  734
KRCV-2/590-1186     703 -----------------EHPNFDSP--------DEFGK------  715
MYBV-1/565-1189     679 -----------------LLPRYKKA--------ENFLA------  691
KKCBV/566-1189      681 -----------------LLPRFKDA--------AEFIR------  693
SGFV/639-1236       754 -----------------CLGKFRNG--------TEFAD------  766
KRTGV/571-1188      687 -----------------LLNRFNSA--------KEFFN------  699
DeMAV/591-1210      707 -----------------LLGRFQSG--------EEFGR------  719
PBJV/596-1189       714 -----------------LLPCIESP--------EEYCN------  726
RtClonAV/431-1533   616 DVGTPTDSVRETWTVSQALARLDTND------AKKFCDIRDLLV  653
RtMrufAV/433-1634   659 DVGSVSSEVVEHWTVSQILSRTGLPD------CDKFASVVDLAH  696
RtEiAV/350-1520     610 LENEPLEDTSIQWTTSQILNRPGLAA------DSLFLDLVTVFE  647
RtMcAV/428-1521     565 -----ASPVLEKWTVAQIYARYECCK--SDKSYGSFLSYHQLLQ  601
PRRSV-2/434-1579    580 -----ASPVTTVWTVSQFFARHSGGNHPDQVRLGKIISLCQVIE  618
PRRSV-1/426-1463    569 ----------EFWTLDKMLTSPSPER-------SGFSSLYKLLL  595
RtClanAV/403-1409   545 ----------NDWTVYRLRHTPGEHL-------KDATALTDIFR  571
LDV/387-1287        546 AMGIP----KDDWVLL-----------------------AELMT  562
APRAV/407-1329      566 STGLV-------------F---------RRIRYDEFRPK-----  582
OSV/389-1122        509 -----------------IYYKIKAG--------ATVSS---LLS  524
EAV/267-832             --------------------------------------------
WPDV/466-1096           --------------------------------------------

ZMV/612-1219        736 ----------------L------------------A--------  737
FSVV/616-1218       740 ----------------L------------------G--------  741
SHEV/632-1235       754 ----------------L------------------G--------  755
KRCV-1/609-1199     735 ----------------L------------------A--------  736
KRCV-2/590-1186     716 ----------------V------------------V--------  717
```

```
MYBV-1/565-1189     692 ----------------H------------------L-------- 693
KKCBV/566-1189      694 ----------------M------------------A-------- 695
SGFV/639-1236       767 ----------------T------------------V-------- 768
KRTGV/571-1188      700 ----------------V------------------T-------- 701
DeMAV/591-1210      720 ----------------V------------------L-------- 721
PBJV/596-1189       727 ----------------V------------------V-------- 728
RtClonAV/431-1533   654 ----GLFAASGVTQQEV-DDAINASIASCSNLSECCAVLEKSLC 692
RtMrufAV/433-1634   697 ----ALFTMTGATPEEI-NAAIDATIVKCASVEECCAAVQKCLK 735
RtEiAV/350-1520     648 ----AAFAPAGASQAEV-HAAIDATINGAKSYSECLQRLLPVAQ 686
RtMcAV/428-1521     602 NTMCGDPKKGRVTEEEL-NDAIDQLIDGSGDIKECLARLTKVRA 644
PRRSV-2/434-1579    619 DCCCSQNKTNRVTPEEV-AAKIDLYLRGATNLEECLARLEKARP 661
PRRSV-1/426-1463    596 E---VVPQKCGATEGAF-IYAVERMLKDCPSSKQAMALLAKIKV 635
RtClanAV/403-1409   572 SCFS--------EEQDVVDQHVNSFVR-GLDLDQTMAALRGVRV 606
LDV/387-1287        563 --------------------------ENCLTRREVLDKLQRGLR 580
APRAV/407-1329          --------------------------------------------
OSV/389-1122        525 D--------------SV------------------V-------- 528
EAV/267-832             --------------------------------------------
WPDV/466-1096           --------------------------------------------

ZMV/612-1219        738 --------CQLCNYLLE--EPGA----PPPRR------------ 755
FSVV/616-1218       742 --------VQLCNFLLK--EPGA----PPPRR------------ 759
SHEV/632-1235       756 --------IKLCNQLLK--EPGA----VPPRR------------ 773
KRCV-1/609-1199     737 --------SALGE----NGHCKR----VSPRR------------ 752
KRCV-2/590-1186     718 --------QSLAAHFIDQGDEKV----PKIRA------------ 737
MYBV-1/565-1189     694 --------QGLAGTK-AVCHHSR----PTPRA------------ 712
KKCBV/566-1189      696 --------RALLCQN-RVCHKPI----PPVRK------------ 714
SGFV/639-1236       769 --------LGLSKTA-HCCNKRV----PTPRK------------ 787
KRTGV/571-1188      702 --------TDVI--------K------NRSSD------------ 711
DeMAV/591-1210      722 --------VALSGGK-WRGVA------ASISD------------ 738
PBJV/596-1189       729 --------CGLVPRA-SLT-KPR----PVLRQ------------ 746
RtClonAV/431-1533   693 RTAVTKSKCDLTAYF-----PE-----YRPA------------- 713
RtMrufAV/433-1634   736 DVAITKQGFALGNYF-----PD-----YQPK------------- 756
RtEiAV/350-1520     687 GLKVTKDKVSLEAYF-----PK-----YVPV------------- 707
RtMcAV/428-1521     645 P-SLFASSVKLNDFF-----PD-----YVPAAS----------- 666
PRRSV-2/434-1579    662 P-RVIDTSFDWDVVL-----PG-----VEAAT------------ 682
PRRSV-1/426-1463    636 P-SSKAPSVSLDECF-----PTDVLADFEPASQ----------- 662
RtClanAV/403-1409   607 SHG---SANDLAATFKRTEEPEEAVNTYKPGA------------ 635
LDV/387-1287        581 LHAT--------------SKPGSP-ASVSPASSIDFSAAGLLLD 609
APRAV/407-1329      583 --------HAVVPPFVPPGAPKPCRPPPPPPSTPGWSTCSSAA- 617
OSV/389-1122        529 --------MQLAPGP-REGPPPP----PPPPA------------ 547
EAV/267-832             --------------------------------------------
WPDV/466-1096           --------------------------------------------

ZMV/612-1219        756 ------------ATVLKFVGA----------------------- 764
FSVV/616-1218       760 ------------QNVLKAVSA----------------------- 768
SHEV/632-1235       774 ------------ADVLKAVSA----------------------- 782
KRCV-1/609-1199     753 ------------KWYLELLSR----------------------- 761
KRCV-2/590-1186     738 ------------KTLLDIMKR----------------------- 746
MYBV-1/565-1189     713 ------------ATLCKVIDT----------------------- 721
KKCBV/566-1189      715 ------------TALVKLMAT----------------------- 723
SGFV/639-1236       788 ------------QAIMSLLNR----------------------- 796
KRTGV/571-1188      712 ------------VQLLGIVDR----------------------- 720
DeMAV/591-1210      739 ------------SELLRIVDS----------------------- 747
PBJV/596-1189       747 ------------RVLASLAVR----------------------- 755
RtClonAV/431-1533   714 -------------NPCRAVVPVKPVKTVTKET-GWT----PHGL 739
RtMrufAV/433-1634   757 -------------NTCTAIVPVADKVKIKEFVPGSTLREAMAGI 787
RtEiAV/350-1520     708 -------------NSCRAIVPVPAVEYVKDRVLGKGKW------ 732
RtMcAV/428-1521     667 -HTAQAPPVVEQQNVCMALVPVKPTGSIVREAAPAE----RLGC 705
```

```
PRRSV-2/434-1579   683 -----QTIKLPQVNQCRALVPVVTQKS----------------- 704
PRRSV-1/426-1463   663 -ERPQ--------SSGAAVVLCSPDAKEFEEAAPEEVQESGHKA 697
RtClanAV/403-1409  636 ----------AVNTQCMALVPAAKSKVSYN-YFGWKEEELTLEA 668
LDV/387-1287       610 GTESDKEAVVAVNNDCYTVLGF---------------------- 631
APRAV/407-1329     618 -----------STDSVDEFVLVSPNSPVTDNQPEL--------- 641
OSV/389-1122       548 ------------DCHCSA-SF----------------------- 555
EAV/267-832            --------------------------------------------
WPDV/466-1096          --------------------------------------------

ZMV/612-1219       765 --------ALKSEP------------------------------ 770
FSVV/616-1218      769 --------AINNKP------------------------------ 774
SHEV/632-1235      783 --------SINQKP------------------------------ 788
KRCV-1/609-1199    762 --------KTA-TD------------------------------ 766
KRCV-2/590-1186    747 --------GRQ-L------------------------------- 750
MYBV-1/565-1189    722 --------PPC-KP------------------------------ 726
KKCBV/566-1189     724 --------TPS-TA------------------------------ 728
SGFV/639-1236      797 --------PNC-VP------------------------------ 801
KRTGV/571-1188     721 --------QGN-PS------------------------------ 725
DeMAV/591-1210     748 --------AAA-TP------------------------------ 752
PBJV/596-1189      756 --------PAN-EP------------------------------ 760
RtClonAV/431-1533  740 ------DVRPQQVSVPLSAETVMNFTYPGCADA---HYAITGVC 774
RtMrufAV/433-1634  788 ------G-TYVEKEVPVTVDRVRHGLCPGGEQA---DWAMGDIC 821
RtEiAV/350-1520    733 --------EVVGKPVLLTLENVMNCSFPGSYET---YSAICSAK 765
RtMcAV/428-1521    706 ------KDRLKNVSVPLSVQSVMDFTYPNCHDV---HYRITEVL 740
PRRSV-2/434-1579   705 ---------LDNNSVPLTAFSLANYYYRAQGDEVRHERLTAVL 739
PRRSV-1/426-1463   698 VHSALLAEGPNNEQVQVVA--GEQLKLGGCGLAVGNAHE--GA- 736
RtClanAV/403-1409  669 IRSGILGRDQTNRE------------------------YLES-- 686
LDV/387-1287           --------------------------------------------
APRAV/407-1329     642 ------------EEVDLS--SPITFGAPAVDEL----------- 660
OSV/389-1122       556 -------------P------------------------------ 556
EAV/267-832            --------------------------------------------
WPDV/466-1096          --------------------------------------------

ZMV/612-1219       771 -----------------------------------------AQL 773
FSVV/616-1218      775 -----------------------------------------THL 777
SHEV/632-1235      789 -----------------------------------------IRS 791
KRCV-1/609-1199    767 -----------------------------------------VPL 769
KRCV-2/590-1186        --------------------------------------------
MYBV-1/565-1189    727 -----------------------------------------ANV 729
KKCBV/566-1189     729 -----------------------------------------AKV 731
SGFV/639-1236      802 -----------------------------------------CIA 804
KRTGV/571-1188     726 -----------------------------------------ASV 728
DeMAV/591-1210     753 -----------------------------------------AKV 755
PBJV/596-1189      761 -----------------------------------------AKV 763
RtClonAV/431-1533  775 RRISLGIREKYGFGKRHPTDPWYPDTPQDLTDLIDKAEEDFTQM 818
RtMrufAV/433-1634  822 RKIRDEMRNTYGYGW-DPVYRWYPDAPADLLDIIEKAETDFLAM 864
RtEiAV/350-1520    766 QRIQNALRDKYGYGKVNPADKWYPDMPDSLVDVVLQAEDDFLKV 809
RtMcAV/428-1521    741 RRAEWCLLARYGQ--------VYQYAPTSFRDMIDECKSELLAI 776
PRRSV-2/434-1579   740 SKLEKVVREEYGLMPTEP--GPRPTLPRGLDELKDQMEEDLLKL 781
PRRSV-1/426-1463   737 -LVSAGLINLVG-GNLSPSDPMKE-------NMLNSREDEPLDL 771
RtClanAV/403-1409  687 --IHSQL---ATRAW---VDHPYPYWFYDYLQIIDHYLDEKVRV 722
LDV/387-1287       632 -------------DKNSATKSEQELATGLFSELVEPMETSTSKH 662
APRAV/407-1329     661 ---QGARVSVYN-----------DDQVRELAELYVSSVKQGLKI 690
OSV/389-1122       557 -----------------------------------------LDF 559
EAV/267-832            --------------------------------------------
WPDV/466-1096      484 --------------TV----KDSAIWVARFA---TLMKTELLDY 506

ZMV/612-1219       774 FG--QK------------------------------------KK 779
```

```
FSVV/616-1218       778 GI--IK------------------------------------PN 783
SHEV/632-1235       792 LE--PA------------------------------------VK 797
KRCV-1/609-1199     770 VE--SS-------------------------------------- 773
KRCV-2/590-1186     751 -----K-------------------------------------- 751
MYBV-1/565-1189     730 SA--SKLIS----------ID---------------------KI 740
KKCBV/566-1189      732 PP--SEVRS----------IL---------------------KP 742
SGFV/639-1236       805 PP--SQVRT----------VD---------------------PS 815
KRTGV/571-1188      729 PH--SYVTA----------VT---------------------PN 739
DeMAV/591-1210      756 PA--SYVTT----------LP---------------------VK 766
PBJV/596-1189       764 PC--SYVRE----------IH---------------------SK 774
RtClonAV/431-1533   819 AG--AQLSL-ENMIWFCQEFDVERHVASY-TWTPCDAPP--EVP 856
RtMrufAV/433-1634   865 CD--AQASL-AGMKWFADNFDVAAHVANY-SWVPQIVTKQPKVV 904
RtEiAV/350-1520     810 VG--AQGTL-ERMTWMAQTFPATEWADRY-VWIADEQPVAVQQP 849
RtMcAV/428-1521     777 AD--RQCTL-EMMANVLEDIDITAFTAKY-KWTPENEP-APRLV 815
PRRSV-2/434-1579    782 AN--AQTTS-DMMAWAVEQVDLKTWVKNYPRWTPPPPP--PKVQ 820
PRRSV-1/426-1463    772 S---------------------------------QPAPASTTTL 782
RtClanAV/403-1409   723 CG--SAARAIDMLRVFSE--DVESLVRMYPIPTPK-------VV 755
LDV/387-1287        663 ES--RK-IL-------------------------EA---ASRAL 675
APRAV/407-1329      691 CLRDSQVQL----------------VESYLSA----------KA 708
OSV/389-1122        560 G-------D----------IK---------------------VN 565
EAV/267-832             --------------------------------------------
WPDV/466-1096       507 IGRDLP-----ADLWLTDDMIVKH--TPYA------------VV 531

ZMV/612-1219        780 PTPKLVATPASVVTSEAPASD--P-------------------- 801
FSVV/616-1218       784 PAA--LTGKA---ESKAKTAN--P-------------------- 800
SHEV/632-1235       798 PS------------APTPAVQ--P-------------------- 807
KRCV-1/609-1199     774 GPSA-----PPLIEGPAV-------------------------- 786
KRCV-2/590-1186     752 GEEN-----QPLLPQPAEEEKPKP-------------------- 770
MYBV-1/565-1189     741 ATV-DAIKPQPAKIAPVPPPR----------------------- 760
KKCBV/566-1189      743 QRPTPDPRTKSTKAAPAPPPA----------------------- 763
SGFV/639-1236       816 QPA------APLPPVPRPRKR----------------------- 830
KRTGV/571-1188      740 SEADFI---KFSKPAPAPRKR----------------------- 757
DeMAV/591-1210      767 DEKDMA---QNAIPTTQHK------------------------- 782
PBJV/596-1189       775 SDLNDC---IPPKPNEAPC------------------------- 790
RtClonAV/431-1533   857 PDSQVQRRRHTVDA----------------PTGSVAVVAAPAVE 884
RtMrufAV/433-1634   905 KAKQPDRRRTP---------------------TPVAPAPPAEPE 927
RtEiAV/350-1520     850 PKKN--RRAAQPAV----------------AAEAAPPAQEVVEE 875
RtMcAV/428-1521     816 ETRQQRRR-------P--QTL-------R--STASEPAKKKVEE 841
PRRSV-2/434-1579    821 PRKTKPVKSLPERK-PVPAPR-------RKVGSDCGSPVSLGGD 856
PRRSV-1/426-1463    783 VREQTPDNPGSDAG-ALPVTV-------R----EFVPTGPILCH 814
RtClanAV/403-1409   756 KIDKAPRPASPKVK-QAPRPRPEPHNRLVWVSSDECVAYRPMAD 798
LDV/387-1287        676 KSAKPKRKRNKKKKTSSPT---------------PTPPETPTRE 704
APRAV/407-1329      709 SEGQVVGGEEPKCGTPCPQPE----KLSKWPS------------ 736
OSV/389-1122        566 PQVFHTRASQPKCLATSMESRPASPVQ----------------- 592
EAV/267-832             --------------------------------------------
WPDV/466-1096       532 T------------------------------------------- 532

ZMV/612-1219        802 ----------PA-------TSAQSAS------------------ 810
FSVV/616-1218       801 ----------PP-------P----KE------------------ 805
SHEV/632-1235       808 ----------TA-------PAHDGKK------------------ 816
KRCV-1/609-1199     787 ----------LP-------ISTSSNA------------------ 795
KRCV-2/590-1186     771 ----------TP-------RLRSQAS------------------ 779
MYBV-1/565-1189     761 -----------K-------ARKVSAD------------------ 768
KKCBV/566-1189      764 -----------P-------ASPP--------------------- 768
SGFV/639-1236       831 -----------K-------AAAQQVS------------------ 838
KRTGV/571-1188      758 -----------K-------AATPLAA------------------ 765
DeMAV/591-1210      783 -----------D-------TAPSVAE------------------ 790
PBJV/596-1189       791 -----------P-------ASTDAIE------------------ 798
RtClonAV/431-1533   885 VPDSWFDLGDGG-------------ASQPLPLDETLG-TLSS 913
```

```
RtClonAV/431-1533   885  VPDSWEDLCDGG---------------ASQPLPLDFILG-ILSS   912
RtMrufAV/433-1634   928  VPDSWEDLATEDQPVDLSPAPAMVAGGNPQFAPLTFGAK-EASW   970
RtEiAV/350-1520     876  VPESWEELADPV---DLSSR------SCGHLPPLLFGAP-TV--   907
RtMcAV/428-1521     842  VPECWESLADSE-PCDLSAVSKGGQ-------RAKFANVSTEEN   877
PRRSV-2/434-1579    857  VPNSWEDLAVSS-PFDLPTPPEPAT-------P-----------   881
PRRSV-1/426-1463    815  VEHCGTESGDSSSPLDLSD-------------------------   833
RtClanAV/403-1409   799  VPDSWDDED---VPLDLSSRSSRSSSS-----------------   822
LDV/387-1287        705  VPGAIEVVSGDE--------------------------------   716
APRAV/407-1329      737  -------ETGTC------SSPTPGVD------------------   749
OSV/389-1122        593  -SGTVQELAKTG-------STTPGKS------------------   610
EAV/267-832              --------------------------------------------
WPDV/466-1096            --------------------------------------------

ZMV/612-1219        811  ---SNADTTPLLS--------AQE-Q-PA------------A--   827
FSVV/616-1218       806  ---KDSDKVPLLS--------AEA-SSPT------------E--   823
SHEV/632-1235       817  ---EHNEKTPLFS--------AEA-STPA------------T--   834
KRCV-1/609-1199     796  NNSNSSASVPAQD-------SACP-EQQD------------K--   817
KRCV-2/590-1186     780  DKSTSS-SQSANA-------------TIT------------S--   795
MYBV-1/565-1189     769  --ASPNTE---AD-------SQPV--TPQ------------L--   784
KKCBV/566-1189      769  --------------------ETPP--DPD------------L--   776
SGFV/639-1236       839  --KVPSEQDPSLA-------HDPP-EKPD------------S--   858
KRTGV/571-1188      766  ---VVTEDQPQSS-------TAPP-EQPK------------E--   784
DeMAV/591-1210      791  --VKVPDAAPSVE-------TQPP-QTPA------------T--   810
PBJV/596-1189       799  --KAQAAQEIARD-------TVAP-DKP----------------   816
RtClonAV/431-1533   913  VSAEPVQCEPLDLSTSGSVKFGPPASQCAG---------ITS--   945
RtMrufAV/433-1634   971  GSECESIDQPLDLSVKGSIKFGPPSSQNSV---------CLT--  1003
RtEiAV/350-1520     908  ASNEQPDDQPLDLSAAGSIKFGPPPSQDEV---------EVC--   940
RtMcAV/428-1521     878  DVEIPVVEQPLCLKA-GTT-LG-PASAA-P-PATLRPQHEVS--   914
PRRSV-2/434-1579    882  SSELVIVSSPQCIFR-PATPLSEPAPIPAPRGTVSRPVTPLSEP   924
PRRSV-1/426-1463    834  ---AQTLDQPLNLSLAAW-PVRATASDPGW-------VHGRREP   866
RtClanAV/403-1409   823  ---CTLAPQPIAAVV-----APPPTPVC----------------   842
LDV/387-1287        717  ---EAGACESATIVPDKAQARPPPRPKRQ---------------   742
APRAV/407-1329      750  ----SSDTVPLTQN-----RFEVPKQRPTPMPRGIRT--GVGSS   782
OSV/389-1122        611  DNT----SQSSTG-------ATPPTGEPAVLP--------VT--   633
EAV/267-832              --------------------------------------------
WPDV/466-1096            --------------------------------------------

ZMV/612-1219        828  ----------DD-----------------PAPSPKLV-------   837
FSVV/616-1218       824  ----------SD-----------------TTPEVKVEQ-----A   835
SHEV/632-1235       835  ----------DQ-----------------ATPAACDTTPAAPPA   851
KRCV-1/609-1199     818  ----------DG--------------------------------   819
KRCV-2/590-1186     796  ----------EG--------------------------------   797
MYBV-1/565-1189     785  ----------GGVNN-------------ETSDLPVPPDTTNPPQ   805
KKCBV/566-1189      777  ----------SDVEL-------------GQNSAAGVPDECAPPL   797
SGFV/639-1236       859  ----------VR--------------------------------   860
KRTGV/571-1188      785  ----------SST-A-------------QPAKAEKVNDPSPPPS   804
DeMAV/591-1210      811  ----------ASNAA-------------APLAAENTNNNAAP--   829
PBJV/596-1189            --------------------------------------------
RtClonAV/431-1533   946  ----------A------------------DLG-PLAPPECGSVL   960
RtMrufAV/433-1634  1004  ----------DM-ESV-------------VLEDPAPALLADVPL  1023
RtEiAV/350-1520     941  ----------DF-ESV------------------LSDPVVAQPL   955
RtMcAV/428-1521     915  ----APT---DA-SSD-------------WTTTSASSALQDEPL   937
PRRSV-2/434-1579    925  IPVPAPRRKFQQ-VKR-------------LSSAAAIPPYQDEPL   954
PRRSV-1/426-1463    867  VFV-KPRNAFSDGDSALQFGELSESSSVIEFDRTKDAPVVDAPV   909
RtClanAV/403-1409   843  --------EFAQAEQV------------------TPPTICAAEI   860
LDV/387-1287        743  --------ALKKAEQGFILK------DIIWNPTE-SGVKCLTIV   771
APRAV/407-1329      783  KPVPAPRTKLPRAA-------------------PESSRPCITPL   807
OSV/389-1122        634  ----------DCTAST---N--------SSAPSGASKRGCCDPA   656
EAV/267-832
```

```
EAV/267-832              --------------------------------------------
WPDV/466-1096        533 ----------------------------------RDQMHCANVL  542

ZMV/612-1219             --------------------------------------------
FSVV/616-1218        836 P-------------------------------------------  836
SHEV/632-1235        852 A-------------------------------------------  852
KRCV-1/609-1199      820 ----SSTA------------------------------------  823
KRCV-2/590-1186      798 ----PAVT------------------------------------  801
MYBV-1/565-1189      806 ATAT----------------------------------------  809
KKCBV/566-1189       798 ASETPEVAA-----------------------------------  806
SGFV/639-1236            --------------------------------------------
KRTGV/571-1188       805 D-EKDA--------------------------------------  809
DeMAV/591-1210           --------------------------------------------
PBJV/596-1189            --------------------------------------------
RtClonAV/431-1533    961 DLSGSSTTVFDLTQRNESPT------------------------  980
RtMrufAV/433-1634   1024 DLSCSSVTVFDAAASNSSPA------------------------ 1043
RtEiAV/350-1520      956 DLSGSSVTEFRAPVAKASSA------------------------  975
RtMcAV/428-1521      938 DLSGSSQTEWMIVSQPTFVPKTRPNTPV----ADEVFDDV-PLD  976
PRRSV-2/434-1579     955 DLSASSQTEYEASPPA--PPQSGGVLGVEGHEAEETLSEISDMS  996
PRRSV-1/426-1463     910 DLTTSN--------------------------------------  915
RtClanAV/403-1409    861 VASLIAQ-------------------------------------  867
LDV/387-1287         772 ----EDVRAFLK---S--ITPPGGALGTR---------------  791
APRAV/407-1329       808 VQASPSDGAA----------------------------------  817
OSV/389-1122         657 AIVLSAEGE-----------------------------------  665
EAV/267-832              --------------------------------------------
WPDV/466-1096        543 GVMINNRGHWKCRLPTYRVAPE----------------------  564

ZMV/612-1219         838 -------------------------------------QE-----  839
FSVV/616-1218        837 -------------------------------------DP-----  838
SHEV/632-1235        853 -------------------------------------DI-----  854
KRCV-1/609-1199      824 ----------------------------------P--AD-----  826
KRCV-2/590-1186      802 ----------------------------------S--GE-----  804
MYBV-1/565-1189      810 ---------------------------------SP--QE-----  813
KKCBV/566-1189       807 ---------------------------------PP--QS-----  810
SGFV/639-1236            --------------------------------------------
KRTGV/571-1188       810 --------------------------------------H-----  810
DeMAV/591-1210           --------------------------------------------
PBJV/596-1189            --------------------------------------------
RtClonAV/431-1533    981 ----PSSPASSASSAVLPVERPPKSAQALIDAQGDMCRTLT--- 1017
RtMrufAV/433-1634   1044 ----QSPPESVFGDEEKQVIRPPKSAQALIDAGGDLAKHLI--- 1080
RtEiAV/350-1520      976 ----PSTT-------STVAKRPAKSAQALIDAKGPLSGHLQ--- 1005
RtMcAV/428-1521      977 LSSKPG-SSASSVVSEPVVMRPKNSAQAIIDANGPLSSHLA--- 1016
PRRSV-2/434-1579     997 GNIKPASVSSSSSLSSVRITRPKYSAQAIIDSGGPCSGHLQ--- 1037
PRRSV-1/426-1463     916 -----EALSVVDPFEFAELKRPRFSAQALIDRGGPLADVHA---  951
RtClanAV/403-1409    868 -----TTP-------------KKLTADEWSEQPTRFGAAIA---  890
LDV/387-1287         792 ---------------------------------ARITAHIVEQF  802
APRAV/407-1329       818 -------EQVTCGSEQPVVEEAKAAPVDLLETAGATSKTLLERI  854
OSV/389-1122         666 ---------------------------------QPVDTE-----  671
EAV/267-832              --------------------------------------------
WPDV/466-1096        565 ----TACSGLGCVHVEPRIERFPT--------------------  584

ZMV/612-1219         840 -IEGTV---------A------RPKSRFRPPDWQALRRYI----  863
FSVV/616-1218        839 -VLNDL---------A------TPKPRFWPPDWTKFRRDV----  862
SHEV/632-1235        855 -GLSDV---------G------KPRARL-RVDWTSFRRKA----  877
KRCV-1/609-1199      827 -DSGTV------------------PVRLKDRVKHW---------  842
KRCV-2/590-1186      805 -EIKTV---------LDDLLNSSQPRGKLATLKED---------  829
MYBV-1/565-1189      814 -PTK------------------SSPITWHDRLLQGT--------  830
KKCBV/566-1189       811 -KIRRT---------V------ADAHYFMTTSLNNA--------  830
SGFV/639-1236        861 ----------------------PPKLGYLDRAWNNMI-------  875
```

```
KRTGV/571-1188     811  -GIKSR---------Y------KTARTWCARAC---R-------   828
DeMAV/591-1210     830  -GTGPP---------P------KRKLTWRERGNNYLA-------   850
PBJV/596-1189      817  ----------------------VGRAPFLLRATSYLQ-------   831
RtClonAV/431-1533  1018 -EIKFRARNMCLAACDPTRLTDPATEAWVNCMWDRLDLLTWKNK  1060
RtMrufAV/433-1634  1081 -IIKKRAMDMCRQACDPNYVDHPSCVIWCESMWNRLDMLTWHNK  1123
RtEiAV/350-1520    1006 -QIKDNVRKICIAATDPNFETHPQTQAWLDAMWNRVDLLTWKNT  1048
RtMcAV/428-1521    1017 -AIKRNVRKVCQQACDPNSLNEPETKDWLDHMWERVDMLTWHNT  1059
PRRSV-2/434-1579   1038 -EVKETCLSVMREACDATKLDDPATQEWLSRMWDRVDMLTWRNT  1080
PRRSV-1/426-1463   952  -KIKNRVYEQCLQACEPGSRATPATREWLDKMWDRVDMKTWRCT   994
RtClanAV/403-1409  891  -HLKASLIDRLVRAHDPSLKDHPAVQLWLVKVWQLINAQAASAE   933
LDV/387-1287       803  HVIRESTPELVL--------AHAEHQA--KNMHE----LLLSEK   832
APRAV/407-1329     855  KALRVSA-------------------------------------   861
OSV/389-1122       672  -ALRDLLIE--DQEHPSNILSNLGKYLWKGSKTPDYSRLV----   708
EAV/267-832             --------------------------------------------
WPDV/466-1096      585  -----------------------IKYDWVFQNSARIESQV----   601

ZMV/612-1219            --------------------------------------------
FSVV/616-1218           --------------------------------------------
SHEV/632-1235           --------------------------------------------
KRCV-1/609-1199         --------------------------------------------
KRCV-2/590-1186         --------------------------------------------
MYBV-1/565-1189         --------------------------------------------
KKCBV/566-1189          --------------------------------------------
SGFV/639-1236           --------------------------------------------
KRTGV/571-1188          --------------------------------------------
DeMAV/591-1210          --------------------------------------------
PBJV/596-1189           --------------------------------------------
RtClonAV/431-1533  1061 SKYQAAFQLGDLS-MLPGMILETPPPYPCPVMLPLGS-PPRSAT  1102
RtMrufAV/433-1634  1124 SKYQASYQLATFEQFLPRMIQETPPPHPCPIMWEPAS-PPRSKT  1166
RtEiAV/350-1520    1049 SKYQAAYQLAPVSSYLPKMIMETPPPYPCPIVLTPPD-PPKSET  1091
RtMcAV/428-1521    1060 SRYQASYQLA-SMDYLPKMILETPPPKPCEVVFAPADTPSQSLQ  1102
PRRSV-2/434-1579   1081 SVYQAICTLDGRLKFLPKMILETPPPYPCEFVMMPHT-PAPSVG  1123
PRRSV-1/426-1463   995  SQFQAGRILA-SLKFLPDMIQDTPPPVPRKNRASDNA-GLKQLV  1036
RtClanAV/403-1409  934  P-WATQRFLNKQFGQINSWLRKVPPPEHLSK-----------KE   965
LDV/387-1287       833  ----AKLILGIGEDTLKKLVS-SQRSLPRSIGFG----------   861
APRAV/407-1329     862  -----ALRLS----------HDGQRPAADA--------GDANKG   882
OSV/389-1122            --------------------------------------------
EAV/267-832        359  --------------------MSGPPPAPVSAAVLDHILEA----   378
WPDV/466-1096      602  ---SEQDLLN--EAFIGGKVITD-QCLLSEITLTPRSSPPPQEP   639

ZMV/612-1219       864  -------------T------TTC--------------------A   868
FSVV/616-1218      863  -------------T------TAC--------------------A   867
SHEV/632-1235      878  -------------T------QAC--------------------A   882
KRCV-1/609-1199         -------------------------------------------A   843
KRCV-2/590-1186         -------------------------------------------I   830
MYBV-1/565-1189         -------------------------------------------L   831
KKCBV/566-1189          -------------------------------------------F   831
SGFV/639-1236      876  ----------------------A--------------------R   877
KRTGV/571-1188     829  ----------------------D--------------------A   830
DeMAV/591-1210     851  ----------------------R--------------------L   852
PBJV/596-1189      832  ----------------------Q---------------------   832
RtClonAV/431-1533  1103 PESD--VTLRTLSEHPPCPPGACTEKDAS----------APALA  1134
RtMrufAV/433-1634  1167 PSVD--LTIRSGVTTPRSRASSLRQEEQKIDAAG-VADVAPTSE  1207
RtEiAV/350-1520    1092 ESVD--LTIRSRVSSKPATPTPPPQMEE-----------VPLCE  1122
RtMcAV/428-1521    1103 SESD--ITLNDLGSMS-----------------GSICSVTPS-Q  1126
PRRSV-2/434-1579   1124 AESD--LTIGSVATEDVPR------ILEKIENVGEMANQGPL-A  1158
PRRSV-1/426-1463   1037 AQWD-----RKLSVTPPPKPVGP--VLDQIV-------PPPTDI  1066
RtClanAV/403-1409  966  SSWET-ISVCSTSSFQDFAA-----------------QAQPDFA   991
```

```
LDV/387-1287       862 -AW---------LSDQQKTADSCGEREFVEVPLKSGAEPTPS--  893
APRAV/407-1329     883 GAHDSDCSGNARRAFLSCKPEDCDQTRPD---------------  911
OSV/389-1122       709 ------------EDERCEVKSC---------------------P  719
EAV/267-832        379 ATF---GNVRVVTPEGQPRPVPAPRVRPSANSSGDVKDPAPVPP  419
WPDV/466-1096      640 RKRKK-RSKQK-PVADPPAPKKLTDQVPEI--KQPVASPV----  675

ZMV/612-1219       869 -H------------------------------------------  869
FSVV/616-1218      868 -N------------------------------------------  868
SHEV/632-1235      883 -H------------------------------------------  883
KRCV-1/609-1199    844 -F------------------------------------------  844
KRCV-2/590-1186    831 -Q------------------------------------------  831
MYBV-1/565-1189    832 -K------------------------------------------  832
KKCBV/566-1189     832 -I------------------------------------------  832
SGFV/639-1236      878 -T------------------------------------------  878
KRTGV/571-1188     831 -I------------------------------------------  831
DeMAV/591-1210     853 -H------------------------------------------  853
PBJV/596-1189          --------------------------------------------
RtClonAV/431-1533 1135 DSEDS-------PP--PAP-------------------------  1144
RtMrufAV/433-1634 1208 DEE-K-------LA--PEPQLAQPATLAGKWRNIFRRDPA---A  1238
RtEiAV/350-1520   1123 DPEDL---------------------------------------  1127
RtMcAV/428-1521   1127 LHEKLAQ--MEQAP--PG-------P------SKDLAAPK----  1149
PRRSV-2/434-1579  1159 FSEDKPV--DDQLV--NDPRISSRRP------DESTSAPS--AG  1190
PRRSV-1/426-1463  1067 QQEDVTP--SDGPP--HAPDFPSRVSTGGSWK------------  1094
RtClanAV/403-1409  992 AQAQLGE--ATQTQ--P----------GGVTQNQPTTTHQGDRA  1021
LDV/387-1287       894 -KRDLGVSLGDQLSQDGAPRLSSSTA------------------  918
APRAV/407-1329     912 --------------------------VHGKSSKEPT--------  921
OSV/389-1122       720 -D------------------------------------------  720
EAV/267-832        420 VPKPRTKLATPNPTQAPIP--APRTRLQGA----STQEPL----  453
WPDV/466-1096      676 ----------------PAPRLHKPQPIKPVVPPVP--APR----  697

ZMV/612-1219       870 -----YCHVGR---------------------------------  875
FSVV/616-1218      869 -----YCHVAR---------------------------------  874
SHEV/632-1235      884 -----YCHVAR---------------------------------  889
KRCV-1/609-1199    845 -----DKYVDF---------------------------------  850
KRCV-2/590-1186    832 -----KGLTQV---------------------------------  837
MYBV-1/565-1189    833 -----NAAAKA---------------------------------  838
KKCBV/566-1189     833 -----AAHDKA---------------------------------  838
SGFV/639-1236      879 -----HKLHNL---------------------------------  884
KRTGV/571-1188     832 -----SALNDP---------------------------------  837
DeMAV/591-1210     854 -----NVIADP---------------------------------  859
PBJV/596-1189      833 ------HAAAA---------------------------------  837
RtClonAV/431-1533 1145 ---AGSDP-VPPPGDSQAPSRSTFC---GGIFASAAST------  1175
RtMrufAV/433-1634 1239 QAEKKSEEVTPPPETTKPPDEGGTPPGNDTTWSRIGAK------  1276
RtEiAV/350-1520   1128 --QQSSKPPDPPGEESKAQADQPN-QPKESLATKAGTW------  1162
RtMcAV/428-1521   1150 ----A--------------------SEDPPPPGKIKGR------  1163
PRRSV-2/434-1579  1191 TGGAGSFTDLPPSD-------G-ADADGGGPFRTVKRK------  1220
PRRSV-1/426-1463  1095 -----------------------------GLMLSGTRL------  1103
RtClanAV/403-1409 1022 QASTSQPAADPPPD--------TSPTGDPGIKGRMGAT------  1051
LDV/387-1287       919 ---CE------------------IKERVPPIKDSGGGL------  935
APRAV/407-1329     922 ----------------KAESVG------NGEAVGEQKWLIRTHN  943
OSV/389-1122       721 -----FDPGDPPDE--------------SSRFDILKEWL-----  740
EAV/267-832        454 -------------------ASAGVASDSAPKWRVAKTVYSS---  475
WPDV/466-1096      698 --TKKQR-PVPAP--RKLPDLV----EKATPVAPAKAR------  726

ZMV/612-1219       876 ---------A-------PDFSRFLPY-LL-------S--LWNSS  893
FSVV/616-1218      875 ---------A-------PNFSRFLPY-LL-------S--LWNTT  892
SHEV/632-1235      890 ---------G-------RDFSRCLPY-LL-------S--FWNTT  907
KRCV-1/609-1199    851 ---------N-------RQVSHIKPH-LL-------H--FLHGS  868
```

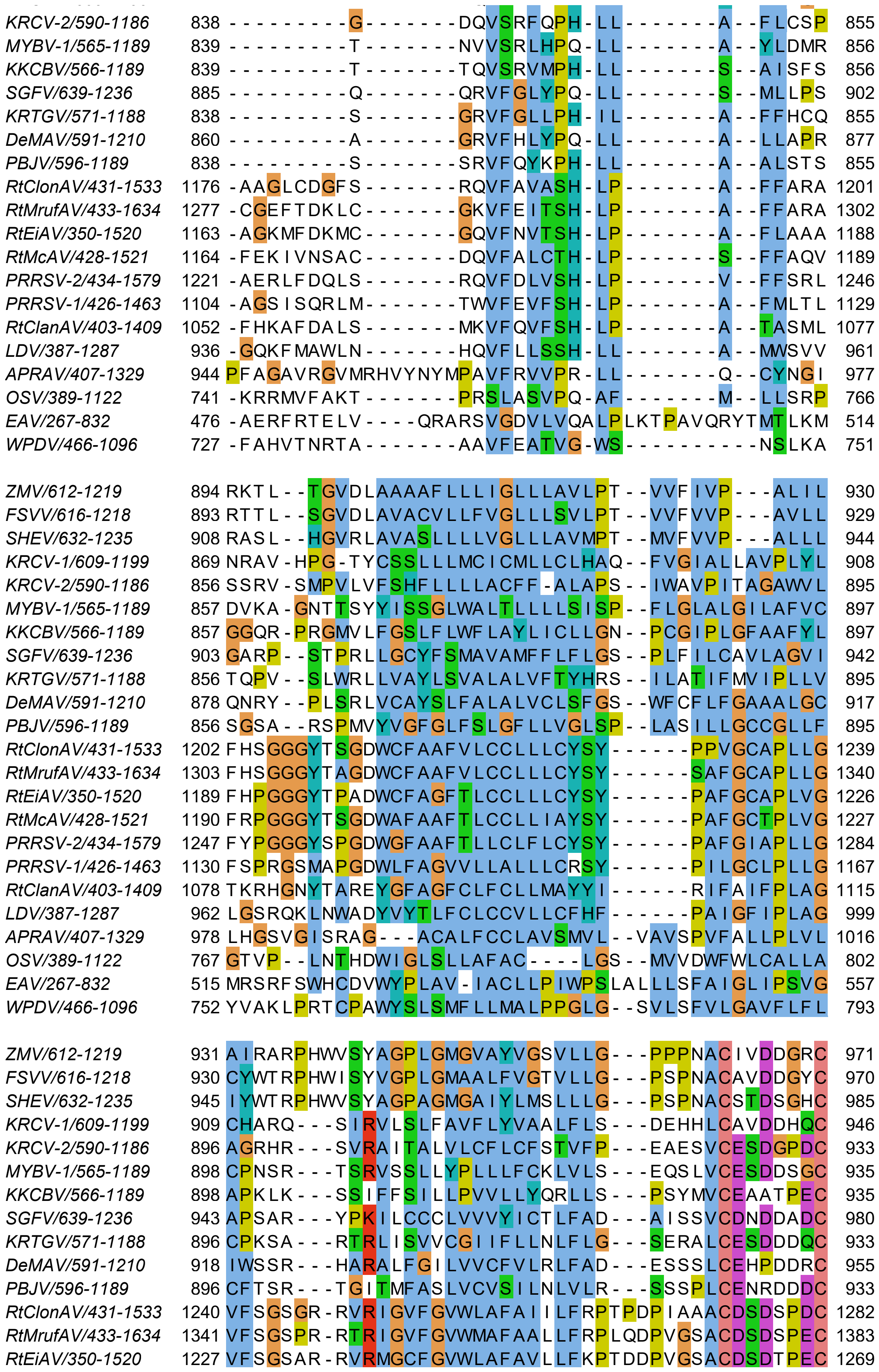

KRCV-2/590-1186 838 ---------G-------DQVSRFQPH-LL-------A--FLCSP 855
MYBV-1/565-1189 839 ---------T-------NVVSRLHPQ-LL-------A--YLDMR 856
KKCBV/566-1189 839 ---------T-------TQVSRVMPH-LL-------S--AISFS 856
SGFV/639-1236 885 ---------Q-------QRVFGLYPQ-LL-------S--MLLPS 902
KRTGV/571-1188 838 ---------S-------GRVFGLLPH-IL-------A--FFHCQ 855
DeMAV/591-1210 860 ---------A-------GRVFHLYPQ-LL-------A--LLAPR 877
PBJV/596-1189 838 ---------S-------SRVFQYKPH-LL-------A--ALSTS 855
RtClonAV/431-1533 1176 -AAGLCDGFS-------RQVFAVASH-LP-------A--FFARA 1201
RtMrufAV/433-1634 1277 -CGEFTDKLC-------GKVFEITSH-LP-------A--FFARA 1302
RtEiAV/350-1520 1163 -AGKMFDKMC-------GQVFNVTSH-LP-------A--FLAAA 1188
RtMcAV/428-1521 1164 -FEKIVNSAC-------DQVFALCTH-LP-------S--FFAQV 1189
PRRSV-2/434-1579 1221 -AERLFDQLS-------RQVFDLVSH-LP-------V--FFSRL 1246
PRRSV-1/426-1463 1104 -AGSISQRLM-------TWVFEVFSH-LP-------A--FMLTL 1129
RtClanAV/403-1409 1052 -FHKAFDALS-------MKVFQVFSH-LP-------A--TASML 1077
LDV/387-1287 936 -GQKFMAWLN-------HQVFLLSSH-LL-------A--MWSVV 961
APRAV/407-1329 944 PFAGAVRGVMRHVYNYMPAVFRVVPR-LL-------Q--CYNGI 977
OSV/389-1122 741 -KRRMVFAKT-------PRSLASVPQ-AF-------M--LLSRP 766
EAV/267-832 476 -AERFRTELV----QRARSVGDVLVQALPLKTPAVQRYTMTLKM 514
WPDV/466-1096 727 -FAHVTNRTA-------AAVFEATVG-WS----------NSLKA 751
ZMV/612-1219 894 RKTL--TGVDLAAAAFLLLIGLLLAVLPT--VVFIVP---ALIL 930
FSVV/616-1218 893 RTTL--SGVDLAVACVLLFVGLLLSVLPT--VVFVVP---AVLL 929
SHEV/632-1235 908 RASL--HGVRLAVASLLLLVGLLLAVMPT--MVFVVP---ALLL 944
KRCV-1/609-1199 869 NRAV-HPG-TYCSSLLLMCICMLLCLHAQ--FVGIALLAVPLYL 908
KRCV-2/590-1186 856 SSRV-SMPVLVFSHFLLLLACFF-ALAPS--IWAVPITAGAWVL 895
MYBV-1/565-1189 857 DVKA-GNTTSYYISSGLWALTLLLLSISP--FLGLALGILAFVC 897
KKCBV/566-1189 857 GGQR-PRGMVLFGSLFLWFLAYLICLLGN--PCGIPLGFAAFYL 897
SGFV/639-1236 903 GARP--STPRLLGCYFSMAVAMFFLFLGS--PLFILCAVLAGVI 942
KRTGV/571-1188 856 TQPV--SLWRLLVAYLSVALALVFTYHRS--ILATIFMVIPLLV 895
DeMAV/591-1210 878 QNRY--PLSRLVCAYSLFALALVCLSFGS--WFCFLFGAAALGC 917
PBJV/596-1189 856 SGSA--RSPMVYVGFGLFSLGFLLVGLSP--LASILLGCCGLLF 895
RtClonAV/431-1533 1202 FHSGGGYTSGDWCFAAFVLCCLLLCYSY------PPVGCAPLLG 1239
RtMrufAV/433-1634 1303 FHSGGGYTAGDWCFAAFVLCCLLLCYSY------SAFGCAPLLG 1340
RtEiAV/350-1520 1189 FHPGGGYTPADWCFAGFTLCCLLLCYSY------PAFGCAPLVG 1226
RtMcAV/428-1521 1190 FRPGGGYTSGDWAFAAFTLCCLLIAYSY------PAFGCTPLVG 1227
PRRSV-2/434-1579 1247 FYPGGGYSPGDWGFAAFTLLCLFLCYSY------PAFGIAPLLG 1284
PRRSV-1/426-1463 1130 FSPRGSMAPGDWLFAGVVLLALLLCRSY------PILGCLPLLG 1167
RtClanAV/403-1409 1078 TKRHGNYTAREYGFAGFCLFCLLMAYYI------RIFAIFPLAG 1115
LDV/387-1287 962 LGSRQKLNWADYVYTLFCLCCVLLCFHF------PAIGFIPLAG 999
APRAV/407-1329 978 LHGSVGISRAG---ACALFCCLAVSMVL--VAVSPVFALLPLVL 1016
OSV/389-1122 767 GTVP--LNTHDWIGLSLLAFAC----LGS--MVVDWFWLCALLA 802
EAV/267-832 515 MRSRFSWHCDVWYPLAV-IACLLPIWPSLALLLSFAIGLIPSVG 557
WPDV/466-1096 752 YVAKLPRTCPAWYSLSMFLLMALPPGLG--SVLSFVLGAVFLFL 793
ZMV/612-1219 931 AIRARPHWVSYAGPLGMGVAYVGSVLLG---PPPNACIVDDGRC 971
FSVV/616-1218 930 CYWTRPHWISYVGPLGMAALFVGTVLLG---PSPNACAVDDGYC 970
SHEV/632-1235 945 IYWTRPHWVSYAGPAGMGAIYLMSLLLG---PSPNACSTDSGHC 985
KRCV-1/609-1199 909 CHARQ---SIRVLSLFAVFLYVAALFLS---DEHHLCAVDDHQC 946
KRCV-2/590-1186 896 AGRHR---SVRAITALVLCFLCFSTVFP---EAESVCESDGPDC 933
MYBV-1/565-1189 898 CPNSR---TSRVSSLLYPLLLFCKLVLS---EQSLVCESDDSGC 935
KKCBV/566-1189 898 APKLK---SSIFFSILLPVVLLYQRLLS---PSYMVCEAATPEC 935
SGFV/639-1236 943 APSAR---YPKILCCCLVVVYICTLFAD---AISSVCDNDDADC 980
KRTGV/571-1188 896 CPKSA---RTRLISVVCGIIFLLNLFLG---SERALCESDDDQC 933
DeMAV/591-1210 918 IWSSR---HARALFGILVVCFVLRLFAD---ESSSLCEHPDDRC 955
PBJV/596-1189 896 CFTSR---TGITMFASLVCVSILNLVLR---SSSPLCENRDDDC 933
RtClonAV/431-1533 1240 VFSGSGR-RVRIGVFGVWLAFAIILFRPTPDPIAAACDSDSPDC 1282
RtMrufAV/433-1634 1341 VFSGSPR-RTRIGVFGVWMAFAALLFRPLQDPVGSACDSDSPEC 1383
RtEiAV/350-1520 1227 VFSGSAR-RVRMGCFGVWLAFAVLLFKPTDDPVGSACDSDTPEC 1269

```
RtMcAV/428-1521    1228 VFSGSAR-RVRMGVFGCWLAFAVVLFQVDSEPVGAACSSDTPEC 1270
PRRSV-2/434-1579   1285 VFSGSSR-RVRMGVFGCWLAFAVGLFKPVSDPVGAACEFDSPEC 1327
PRRSV-1/426-1463   1168 VFSGSLR-RVRLGVFGSWMAFAVFLFSTPSNPVGSSCDHDSPEC 1210
RtClanAV/403-1409  1116 CFMGSSR-IVKLAVFSTWMGFAVLLFAADAEESPLDCDSDPGEC 1158
LDV/387-1287       1000 CVFGSPW-RVRLSVFSVWLCVAVVVFQEVLPEPGSVCSSASAEC 1042
APRAV/407-1329     1017 LCTRVRGRRLCFVISGMALAR-WIQLANTEAAVIQSCHTGAPEC 1059
OSV/389-1122        803 ALLHTPRGVLYAFVGLLG--FAASLLAGRRSLDLSACDAGLETC 844
EAV/267-832         558 N----------------NVVLTALLVSSANYVASMDHQCEGAAC 585
WPDV/466-1096       794 TVSPVPL--VIS-----VTLFSWFVLT---RPHFIQCSSWDREC 827

ZMV/612-1219        972 DTALR--ALSTRFSAGHGHSYTLGPVSFVHVLFKYFVHLEADLH 1013
FSVV/616-1218       971 DSALL--VLAQRFSAGRPVRYSLGSLSFVFILYEYFVYVETDLS 1012
SHEV/632-1235       986 DTALH--ALAARFANRRAGRHSLGPTSTLFVLYEYFVYMEADLS 1027
KRCV-1/609-1199     947 LQFLH--DLQRRFSTSPPRFLTPGPITAGAVFVNYFWGAVVLGY 988
KRCV-2/590-1186     934 RDRLT--ALANRFRGAVVRYVGAGPGTCILLFARSLYYFESAAS 975
MYBV-1/565-1189     936 RDFLL--SVSLRYASAPPRFVTPGPFTAGFAVLRNFVIVTTLVQ 977
KKCBV/566-1189      936 DDYLR--SLVDVFSNPPPKFVTPGPCTLALAIVRSYTRIPSGVY 977
SGFV/639-1236       981 RAFLS--DLGDRYSTNQPVYITPGPATFFLAVSRNFFVVSVALF 1022
KRTGV/571-1188      934 LRAID--HLRLRFSTTAPVYVTPGLLTVGFALLRNFIYTGAASV 975
DeMAV/591-1210      956 HEYLD--SVRGRLSASFRTFITPGLLTVFLAFFRGLYPITSALF 997
PBJV/596-1189       934 VAYLH--QLRDRYDDPPSVYLTPGPFTAFFAFIRWLRFVSLRPL 975
RtClonAV/431-1533  1283 RRVLL--DFERQQPWDPVRSLVVGPLGLGTAILGRLLGGARYFW 1324
RtMrufAV/433-1634  1384 RRLLL--AFEQLQPWKPVRGLVVGPFGLTASLLGKLLGGPRNFW 1425
RtEiAV/350-1520    1270 RQLLL--AFEQRQPWEPVRGLVVGPIGLTASILGKLLGGARSVW 1311
RtMcAV/428-1521    1271 RDLLL--AFEQRQLWNPVRSLILGPWGAVAAILGRILGGPRYFW 1312
PRRSV-2/434-1579   1328 RNILH--SFELLKPWDPVRSLVVGPVGLGLAILGRLLGGARCIW 1369
PRRSV-1/426-1463   1211 HAELL--ALEQRQLWEPVRGLVVGPSGLLCVILGKLLGGSRYLW 1252
RtClanAV/403-1409  1159 RRRLE--YYVDQQPWRVVRGIVVGPVGLATAIFGQLLGGSRYLW 1200
LDV/387-1287       1043 AAALE--RYSGNGVHRPVNHIGVGLVGTVAGFVARVVGGPRHYW 1084
APRAV/407-1329     1060 LELRS--FIAGNILDGPVSVATIGPFGVLGSFLAGVLGGDRYGW 1101
OSV/389-1122        845 RPILQ--GFVLHRASGLDLVPTVGVVGVLVSIIARLVGGSTLVY 886
EAV/267-832         586 LALLEEEHYYRAVRWRPI----TGALSLVLNLLGQVGYVARSTF 625
WPDV/466-1096       828 LATNG---LPMPESIVVMNRGSAGLIGLVIHLFAY-AGMSRRIF 867

                                                            *
ZMV/612-1219       1014 -LLSSVLC-MLDLLLCVLWLFRRRYCWRCFAPCIRTA-PAEIPL 1054
FSVV/616-1218      1013 -LLSTLLC-LCDLVLCTLWVVRRCHCWRCFAPCIRCA-PGEIPL 1053
SHEV/632-1235      1028 -LISSLLC-VFDVVLCCAWLLRRRYCWRCFAPCIRTA-PSEVVL 1068
KRCV-1/609-1199     989 -FLYYLGF-LIDIGLVGLVAGHNRICWRCFRPCIRTA-PEELVL 1029
KRCV-2/590-1186     976 -MLYALLF-IFDLALLCCILSHNRICLRCYGCCIRKA-PEEVVL 1016
MYBV-1/565-1189     978 -YAHFLLL-VFDVCILLLCMCHRSFCLKCYGRCIRLA-PEEIQG 1018
KKCBV/566-1189      978 -FLHILAL-TCDLLIFCLLMYYNSICHKCFSFCIRKA-PEEIQL 1018
SGFV/639-1236      1023 -PLHLLLL-MVDVLLVIGVLCMDGYCFRCFSRCVRKA-PEEVSL 1063
KRTGV/571-1188      976 -YIHCLLL-LADIVLILALMFLNGICLRCFGRCIRTA-PHEVHL 1016
DeMAV/591-1210      998 -VLHYFLL-LLDLVFILALLFVRRICLRCWGRCIRTA-PEEVPL 1038
PBJV/596-1189       976 -LVHCILL-LLDSLLLLLILLSHKVCRHCWRRCIRLA-PEEIVL 1016
RtClonAV/431-1533  1325 FVLLRLCF-LGDLLLAGAYIVSQGRCKRCWGRCVRMA-PSEIPL 1366
RtMrufAV/433-1634  1426 AILLRLMF-MADVIAAGAYIVGQGRCKKCWGRCIRTA-PQEVAM 1467
RtEiAV/350-1520    1312 WILLRLCF-VADVVCAGAYVIGQGRCKKCWGRCIRTA-PSEVAF 1353
RtMcAV/428-1521    1313 HCLLRFGF-LADVLFYCFYIVSQGRCKRCWGQCIRTA-PSEVPF 1354
PRRSV-2/434-1579   1370 HFLLRLGI-VADCILAGAYVLSQGRCKKCWGSCIRTA-PNEVAF 1411
PRRSV-1/426-1463   1253 HVLLRLCM-LADLALSLVYVVSQGRCHKCWGKCIRTA-PAEVAL 1294
RtClanAV/403-1409  1201 SCLLRLSV-AFDLLACVVYVVMQGRCKKCWGMCVRVA-PAEVVM 1242
LDV/387-1287       1085 FYFLRLMV-VLDLGLVFLAVALRGRCKKCFCKCVRVA-PHEVHL 1126
APRAV/407-1329     1102 TLVLRAAF-AVDLALVIIGFISQNRCKRCLGNCIRTVKTAEIPT 1144
OSV/389-1122        887 HLLLRGGV-IVDMLLLGVGYYFSGVCTKCWRPCIRTA-HRDVNL 928
EAV/267-832         626 DAAYVPCT-VFDLCSFAILYLCRNRCWRCFGRCVRVG-PATHVL 667
WPDV/466-1096       868 ATLRVVSVAVDTLALYAAYVLDGVLCFKCFHQCARTQKK-LHSS 910
```

```
                             *
ZMV/612-1219      1055 RAVPLTRVSSTTLLDICDNFSRPP-VDIIKMATGYKGCYCGQLP 1097
FSVV/616-1218     1054 RTVPISRVSNSTLLDICDNFSSPP-VDIIRMATGYKGCYRGSLP 1096
SHEV/632-1235     1069 RTVPLSRVSLSTLLDICDNCARPP-VDVIKMATGYSGCYCGNLP 1111
KRCV-1/609-1199   1030 LTVPASRIHRATVLDVCDNYSNPP-VDIVKQATGYAGCYRGQLP 1072
KRCV-2/590-1186   1017 KTIPLSRISRSTLLDICDSFSRPP-VDIILQATGWRGCYTGCFN 1059
MYBV-1/565-1189   1019 RCVPSSRLSRVSLVDICDVYKAPP-CDIIKMATGYAGCYTGSLS 1061
KKCBV/566-1189    1019 CTVPSSRVSKHTLLDICNNYQRPP-VDVVQMATGYRGCYTGVLN 1061
SGFV/639-1236     1064 LTIPQSRVSRRFLLDICDFYSAPP-VDIIRLATGLNGCFRGDYS 1106
KRTGV/571-1188    1017 CTIPASRVSRATLLDICDTFSAPP-IDIIRMATGYAGCYQGCIN 1059
DeMAV/591-1210    1039 LTIPSSRVTRAFLLDIANTFSSPQ-VDVIRMATGYAGCYDGCVC 1081
PBJV/596-1189     1017 PTIPSSRTSRAVLLDIADAFAPPP-VDVIRLATGYSGCFRGVLP 1059
RtClonAV/431-1533 1367 NVFPFTRATRTSLVDLCNRYCAPKGIDPIFVASGWRGCYSGDSP 1410
RtMrufAV/433-1634 1468 NVFPFTRATRQSLVDICNRFCSPKLIDPIFVATGWRGCYIGQSP 1511
RtEiAV/350-1520   1354 NVFPFTRGTRQSLVDLCDRFCKPKGMDPIQLACGWSGCWSGESP 1397
RtMcAV/428-1521   1355 NVFPFTRATRQSLVTLCDRFSRPKGMDPIHLATGWHGCWTGQSP 1398
PRRSV-2/434-1579  1412 NVFPFTRATRSSLIDLCDRFCAPKGMDPIFLATGWRGCWAGRSP 1455
PRRSV-1/426-1463  1295 NVFPFSRATRVSLVSLCDRFQTPKGVDPVHLATGWRGCWRGESP 1338
RtClanAV/403-1409 1243 KIFPLTRCNKSSLVDLCDRFCAPKLVDPIKLTTGWAGCWSGAAP 1286
LDV/387-1287      1127 RVFPLTKVARPTLEAVCDMYSAPR-VDPILVATGIKGCWQGKVS 1169
APRAV/407-1329    1145 FTVPSTKVATAALVNYCDVVSKEY-PDLVFMLTGVRGCMCGSRT 1187
OSV/389-1122       929 VCVPAAKVSRLTLTTICDLYCAPP-VDFIKLATGYHGCYTGTVS  971
EAV/267-832        668 GS-TGQRVSKLALIDLCDHFSKPT-IDVVGMATGWSGCYTGTAA  709
WPDV/466-1096      911 EKAKNIVVNNTMILQFMDTYAPPP-VDLVKLATGVNGCHCGSKS  953

ZMV/612-1219      1098 CMT-EGIKPVSYSKLDTKKVSPRTVIPVPTTPNEAVKAIAVLNA 1140
FSVV/616-1218     1097 CVT-EGAKPISYSKLDMKKLSPRTVVPFPTTPAEAVKAIHVLNA 1139
SHEV/632-1235     1112 TVT-AGTKPISYSKLDLKKLSPRTVIPIPSTPAEAVKAIHVLNA 1154
KRCV-1/609-1199   1073 SVA-SVCCNVPAAKVDVKKVSAKTVCSIPTSPSEAVKALQVLNA 1115
KRCV-2/590-1186   1060 PVV-DSASPIPVSRVDPKKITSNTICTLPVCPAEAVKAIHVLHN 1102
MYBV-1/565-1189   1062 CVK-ACSSALPVAHIDPKKVSNTTSCTFPTCASEAVKALAVLSA 1104
KKCBV/566-1189    1062 STI-NSNSSIPCTKIDPKKVTNSTVCTMPSCASEAARAICVLSA 1104
SGFV/639-1236     1107 PIG-SSTSVITADKIDVKKVSCRTVCSFPSCPSEAVKVLHVLSV 1149
KRTGV/571-1188    1060 PTG-AAAT-IECARVDPKKVTPSTCASFPSCASEAVKAIHVLSA 1101
DeMAV/591-1210    1082 PTG-TSAN-IAVGKVDVKKVTHKTSCSVPTCPTEAVKALHVLAS 1123
PBJV/596-1189     1060 AVG-VGSGVISCDAIDTKKVRANTLCSMPSTPAEAVKAIHVLSA 1102
RtClonAV/431-1533 1411 IEQ-PSSTPISYVNLDEKKISSRTVVSPPYDPSQAIKCLKVLQA 1453
RtMrufAV/433-1634 1512 IEQ-PSSKPFTYQNLDEKKISATTIVTPPYDPTQAIKCLKVLQA 1554
RtEiAV/350-1520   1398 IEQ-PSSKPISYQALDEKKISASTVVAQPYDPNQAIKCLKVLQA 1440
RtMcAV/428-1521   1399 IEQ-PCDKPITYANLDEKKVSAQTVVAQPYDPNQAVKCLRVLQS 1441
PRRSV-2/434-1579  1456 IEQ-PSEKPIAFAQLDEKKITARTVVAQPYDPNQAVKCLRVLQS 1498
PRRSV-1/426-1463  1339 IHQ-PHQKPIAYANLDEKKMSAQTVVAVPYDPSQAIKCLKVLQA 1381
RtClanAV/403-1409 1287 IEL-ACSKPISYSNLDEKKIGPQTVVPPPLDPTQGIKCLKVLQA 1329
LDV/387-1287      1170 PHQ-VTDKPVSYSNLEEKKISNKTVVPPPTDPQQAVKCLKVLQC 1212
APRAV/407-1329    1188 LSP-TS--STSYKNLDPVKVGPRTVLPWPSNVTQATAALKSVLN 1228
OSV/389-1122       972 CTT-QGKA-IPYAKLDKNKIGNGTLIPLPVTPNQAKDAINVILN 1013
EAV/267-832        710 MER-QCASTVDPHSFDQKKAGATVYLTPPVNSGSALQCLNVMWK  752
WPDV/466-1096      954 FIQWSTARPVAYSRYDPTKSSAETVLPLPKNAEQALRVISHALE  997

ZMV/612-1219      1141 RGV----MARSAH-TVERVDKLP----CKNPFFPY--DLNLKRI 1173
FSVV/616-1218     1140 RGV----MARSPH-TVERVDKLP----CKNPFFPY--DVNSRRI 1172
SHEV/632-1235     1155 RGV----MTPLVH-LVEKVDKLP----CKNPFFPY--DLNNKSV 1187
KRCV-1/609-1199   1116 RGQ----LGYENH-AVEAVAVLP----CANPFFPY--DTSSSKI 1148
KRCV-2/590-1186   1103 RGQ----LAFDRTAKVEKVETLP----CKNPFFPY--DVMSRKI 1136
MYBV-1/565-1189   1105 RGT----LSLGKPPRVVKVEKFP----CKNPFFPY--DLSLTTP 1138
KKCBV/566-1189    1105 RGH----LSRGGLAKVVKVEKLP----CKNPFFPY--DLASSTP 1138
SGFV/639-1236     1150 RGQ----MCAHNEQKVEKVDALP----CKNPLFPY--DLSSKKI 1183
KRTGV/571-1188    1102 RGT----IGPFNNAKVEKVEKLP----FKNPLFPY--DVDNKKV 1135
DeMAV/591-1210    1124 RGT----IAPLNNNKVKKVDALP----CRNPLFPF--DIKNKVI 1157
```

```
PBJV/596-1189      1103 RGQ----LAYQSNCKVEKVEKLP----CRNPIFEY--DVNSRNI 1136
RtClonAV/431-1533  1454 GGA----MAACRVPAVVKLTQVP----FLAPFLPK-LPVNPDAR 1488
RtMrufAV/433-1634  1555 GGA----IAATKVPEVTKIDSVP----FLAPFLPK-LPVNPDVK 1589
RtEiAV/350-1520    1441 GGA----MVAIKVPTVTKVNTVP----FLAPFLPK-CPVNPDVK 1475
RtMcAV/428-1521    1442 GGA----MVAEAVPKVVKVQTVP----FLAPFFPK-VRVDADTK 1476
PRRSV-2/434-1579   1499 GGA----MVAKAVPKVVKVSAVP----FRAPFFPTGVKVDPDCR 1534
PRRSV-1/426-1463   1382 GGA----IVDQPTPEVVRVSEIP----FSAPFFPK-VPVNPDCR 1416
RtClanAV/403-1409  1330 GGA----IVDVAAPKVVKVEEVP----FLAPFFPK-TKVNPESI 1364
LDV/387-1287       1213 GGS----IQDVGVPEVKKVSKVP----YKAPFFPN-VSIDPECY 1247
APRAV/407-1329     1229 GAS----ICSRTPLKVTPAKTVP----FKHETFPD-LPVDPDQE 1263
OSV/389-1122       1014 GGS----FATSNLPPVIKVFSVP----FRSVYFPN-LPVDPSKP 1048
EAV/267-832         753 RPIGSTVLGEQTGAVVTAVKSIS----FSPPCCVSTTLPTRPGV 792
WPDV/466-1096       998 HGVVVFHMGHT-GVDVKRIEAYPASISPLPDFFPDLPYTSTEAT 1040

ZMV/612-1219       1174 VVVDAETMDLFSDLGV-DVSHLVVGDGDFFKVMGVARPPANVAI 1216
FSVV/616-1218      1173 VAVDAETYSLFKDLGL-DLSHLVIGEGDFFKCMGVVRPPAQNAV 1215
SHEV/632-1235      1188 VAVDPDTYSLFSELGL-DLSHLVIGEGDFFKAMGVKRPASSAVV 1230
KRCV-1/609-1199    1149 VTVCAYTYNLFTELGV-DTSLLTIGDGDFFKAMGVEKPKSFCLA 1191
KRCV-2/590-1186    1137 VVVDSATFELFTQLGL-DTSMLVLGQGDFFEAMGVARPTKLAAA 1179
MYBV-1/565-1189    1139 VVVDCATYELFAELGL-NVSHLVIGEGDIFQVMNVKRPTFYEKA 1181
KKCBV/566-1189     1139 VVVDNYTFELLSDLGV-NVSLLTVGEGDFFAAMGVARPSFFEKA 1181
SGFV/639-1236      1184 VPVDSGTYEILSSIGC-DMSHLVIGDGDFFKVMGVPRPSPFTVM 1226
KRTGV/571-1188     1136 VVVDPTTYTLFSELGC-DVSHLVLGSGDFFKAMNTPRPDAFTIL 1178
DeMAV/591-1210     1158 TCVDSDTYSLLSELGC-DLSHLIIGTGDFFQEMGVPRPDYFTVL 1200
PBJV/596-1189      1137 VTVDAKTFELLRDLGC-DTSHLIIGSGDFFEVMGVKRPDRLGVA 1179
RtClonAV/431-1533  1489 IVVDSETFTCALRSGY-DTSNFILGEGNFAELNGLKIQQLQ--- 1528
RtMrufAV/433-1634  1590 IVVDTETFTCAIRSGY-DTSALILGEGDFAKENGMKIQQLQ--- 1629
RtEiAV/350-1520    1476 IVVDPDTFTTAIRCGY-DVSSFVLGEGDFAAANGLKVQQMQ--- 1515
RtMcAV/428-1521    1477 IVVDPPTFSAALRSGY-STSALIIGEGDFAKINGVKVGCYV--- 1516
PRRSV-2/434-1579   1535 VVVDPDTFTAALRSGY-STTNLVLGVGDFAQLNGLKIRQIS--- 1574
PRRSV-1/426-1463   1417 VVVDSDTFVAAVRCGY-STAQLVLGRGNFAKLNQTPPRNSIST- 1458
RtClanAV/403-1409  1365 IVVDVPTYTAALRCGY-DVSQLVLGEGDFAKMNGLVASGPL--- 1404
LDV/387-1287       1248 IVVDPVTYSAAMRGGY-GVSHLIVGTGDFAEVNGLRFVS----- 1285
APRAV/407-1329     1264 IIVDLPTYHLLSTQKC-KMDKVVIGEGDFAAVNGILPGN----- 1301
OSV/389-1122       1049 IVVDQSIFARLSAE---QRNQVILGSGDFVAVNKLMPPELDPVH 1089
EAV/267-832         793 TVVDHALYNRLTASGV-DPALLRVGQGDFLKLNPGFRL------ 829
WPDV/466-1096      1041 LVVDVNLKAALSACGYPDMDKIVVGEGDWLLENEVYPIGVDQKR 1084

ZMV/612-1219       1217 K------------------------------GG            1219
FSVV/616-1218      1216 S------------------------------GG            1218
SHEV/632-1235      1231 R------------------------------GG            1233
KRCV-1/609-1199    1192 A-----------------------LKKRV--GG            1199
KRCV-2/590-1186    1180 A-----------------------LRVRG--G-            1186
MYBV-1/565-1189    1182 R-----------------------LKLTK--GG            1189
KKCBV/566-1189     1182 Q-----------------------LKLLR--GG            1189
SGFV/639-1236      1227 R-----------------------LRACRVVGG            1236
KRTGV/571-1188     1179 K-----------------------LKATRRLGG            1188
DeMAV/591-1210     1201 R-----------------------LKAARIMGG            1210
PBJV/596-1189      1180 L-----------------------MQARRISGG            1189
RtClonAV/431-1533  1529 ----------------------------RPKGG            1533
RtMrufAV/433-1634  1630 ----------------------------RPSGG            1634
RtEiAV/350-1520    1516 ----------------------------RPSGG            1520
RtMcAV/428-1521    1517 ----------------------------APSGG            1521
PRRSV-2/434-1579   1575 ----------------------------KPSGG            1579
PRRSV-1/426-1463   1459 ----------------------------KTTGG            1463
RtClanAV/403-1409  1405 ----------------------------PNKGG            1409
LDV/387-1287       1286 -------------------------------GG            1287
APRAV/407-1329     1302 GKIWANNVITPERAQIPGIN----LVALRNLG-            1329
```

OSV/389-1122	1090	GVLWAEKVLTPARTSWLSASQTQKLCDAGYAGG	1122
EAV/267-832	830	------------------------------IGG	832
WPDV/466-1096	1085	TCRYLSHHVSPG---------------------	1096

Supplementary Figure 3: Alignment of nsp2 across all currently known arteriviruses.

The first residue in the alignment corresponds to the N-terminal border of the PLP2 and the last residue prior to the nsp2/3 cleavage site. Residues were colored according to the Clustal coloring scheme, with conserved positively-charged residues selected for mutagenesis studies indicated by black asterisks.

Supplementary Figure 4

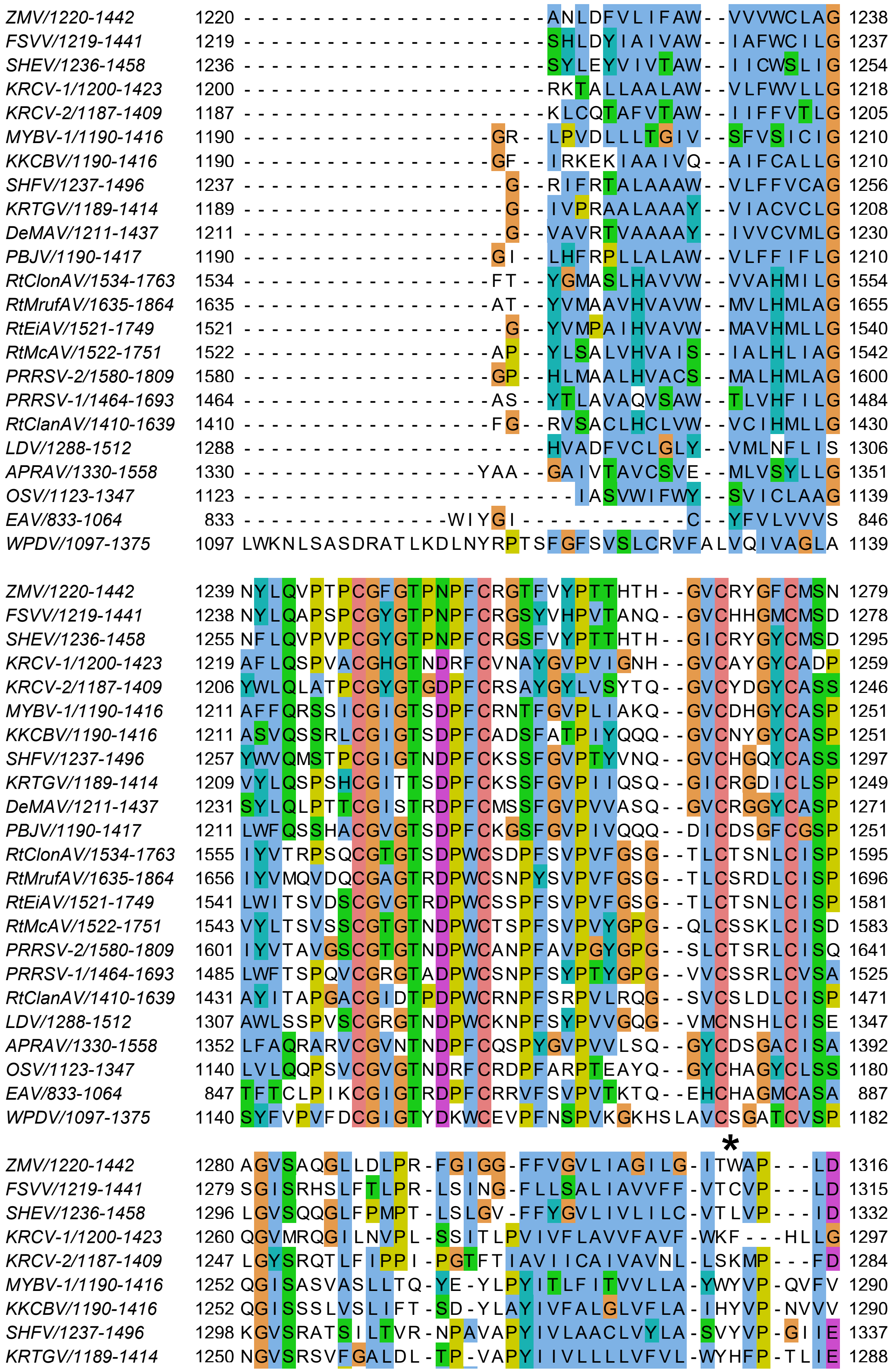

```
                                                                         *
DeMAV/1211-1437      1272 LGISRNTPDLLSL-APAVAPYIAILLLICFLL-WQYVP-TVVE 1311
PBJV/1190-1417       1252 FGVSRSLVHYLTP-DVSVAPYLVLAITVAYLL-LSYVP-GLLE 1291
RtClonAV/1534-1763   1596 SGLTLPLATALRD-FGAREASIVGLVLLAVACVAHRLA-VKAD 1636
RtMrufAV/1635-1864   1697 SGLTLPLSTIIKD-FGAREAGVIGLVIASLAVLAHKMS-MKSD 1737
RtEiAV/1521-1749     1582 AGLTLPLASLVKD-FGAREVGIIGLVLASVACLAHKLA-VKTD 1622
RtMcAV/1522-1751     1584 KGLTLPLIFAASN-LGWSEAGYIALVFASLLFVANRLA-LKAD 1624
PRRSV-2/1580-1809    1642 HGLTLPLTALVAG-FGIQEIALVVLIFVSIGGMAHRLS-CKAD 1682
PRRSV-1/1464-1693    1526 DGVTLPLFSAVAQ-LSGREVGIFILVLVSLTALAHRMA-LKAD 1566
RtClanAV/1410-1639   1472 EGLSLPLQSVLVT-LGIKEWAAVALVITSLLALGQRYA-VQAD 1512
LDV/1288-1512        1348 DGLTSPMVL---S-YSLIDWALMIAVIATVAIFIAKVS-LLVD 1385
APRAV/1330-1558      1393 AGYSVEAPAIFVD-STLLTCCTLGFMAILLFC-AEIVTL--FD 1431
OSV/1123-1347        1181 AGLSTAGSLMPQG-LIGLVQTSLLFVLALAAG-AKLLRLSMVE 1221
EAV/833-1064          888 EGISLDSLGLTQL-QSYWIAAVTSGLVILLV--CHRLAISALD  927
WPDV/1097-1375       1183 DGVFTRDPVFPVAICGLYISA-----LAAAAIVISKLRISVGA 1220

ZMV/1220-1442        1317 ALVM--------STLLVLPSGSLFTALRVVIIVLAAKFMNFRH 1351
FSVV/1219-1441       1316 GVVI--------STLLLLPVNPYCTVFRIVLLVASSAFISFKN 1350
SHEV/1236-1458       1333 GLVV--------SALLVLPPSSYTTLIRICVILLGSKFMTFRN 1367
KRCV-1/1200-1423     1298 YVYS--------LLPFFLPNLPLVVPLRVLICIAGSLRVPLHF 1332
KRCV-2/1187-1409     1285 YFLS--------AVFLLLPTGTYYTLCRIAVFIFGMNHVSCFT 1319
MYBV-1/1190-1416     1291 IGII--------FLNALAPTTPLTSVMRVCAFFVLSTKIDIKL 1325
KKCBV/1190-1416      1291 VAAL--------MLNTLTPVTPFTSFIRVLVMVCSSGYVEFRV 1325
SHFV/1237-1496       1338 VSLL--------VLNALLPAGPAISALRTLVMIIAAPHLSMKY 1372
KRTGV/1189-1414      1289 AGFT--------LLVALLPGSHAVNVLRVGLALAFAPFVSIKV 1323
DeMAV/1211-1437      1312 AIAV--------LIAAAMPSTPLVDAIRVILFFLALPRLSTKV 1346
PBJV/1190-1417       1292 TVCL--------LINAVLPTSPLVTVIRVICFQVALPHLTQRG 1326
RtClonAV/1534-1763   1637 ALAV--------LFSLVAYVYPLASW-ALACFPVLMRWLPLHP 1670
RtMrufAV/1635-1864   1738 AVFV--------LASLFCHVHPLLAW-VVALFPLTLKWFSAHP 1771
RtEiAV/1521-1749     1623 ALLV--------LASILCHAHPMLAW-VVCLYPLALRWCSVSP 1656
RtMcAV/1522-1751     1625 LILV--------LCAMACYVNPMLAW-IVCLYPLALRHFTLHP 1658
PRRSV-2/1580-1809    1683 MLCV--------LLAIASYVWVPLTW-LLCVFPCWLRCFSLHP 1716
PRRSV-1/1464-1693    1567 MLVV--------FSAFCAYAWPMSSW-LICFFPILLKWVTLHP 1600
RtClanAV/1410-1639   1513 ILQV--------MACILTYTFPSLSW-VACLFPLELKWISLHP 1546
LDV/1288-1512        1386 VICV--------FLCLLMYVFPPLSV-IAFAFPFALCKVHLHP 1419
APRAV/1330-1558      1432 IFAP--------LLSIGALWYSPLQF-IAAFTPV-LLRFGGHR 1464
OSV/1123-1347        1222 LGLL--------LLPFCTAVHPTLHF-VTLLGPV-SLWWGVNT 1254
EAV/833-1064          928 LLTL--------ASPLVLLVFPWASVGLLLACSLAGAAVKIQL  962
WPDV/1097-1375       1221 IVTVIGSVLNYCFFPEYCYAGPLIFL-----AQLFMPW-TIQS 1257

ZMV/1220-1442        1352 ICLGL--ALVAPFDPYAAVTGLIILGC--CVLSARYT------ 1384
FSVV/1219-1441       1351 LGIGL--ALIAPLDPTAACAGLIILGC--CYFTGRYT------ 1383
SHEV/1236-1458       1368 LGICL--AVVTPLDPYASLTGAIILAC--CYLSSAFT------ 1400
KRCV-1/1200-1423     1333 NIIHL--AATFYMDPAGGVLTAVIAAA--AWALGRFT------ 1365
KRCV-2/1187-1409     1320 VASWL--AIGLILDINAFFVCAAVAVL--GWIVSN-T------ 1351
MYBV-1/1190-1416     1326 LLLTV--LTTSLIDLPASIICFILFGS--AWVIGKCT------ 1358
KKCBV/1190-1416      1326 LVFAI--ISSALLDPTASIVILILLSL--TWAIGKWT------ 1358
SHFV/1237-1496       1373 IAFFC--CTTAFVDFTSVVVVLTALLV--GWILARYT------ 1405
KRTGV/1189-1414      1324 LVFHI--CTTMLLSPICAFVILVVLLV--AWYVGNQT------ 1356
DeMAV/1211-1437      1347 IGFYL--ASTVLISPAAACISAIGMAC--AWIVGAFT------ 1379
PBJV/1190-1417       1327 IVIFT--VTCALIDIPSLVTICILAIS--AWVLGKYT------ 1359
RtClonAV/1534-1763   1671 FTALWVHFFLVACNPPAGGLALAFSFT--CWILGRYT------ 1705
RtMrufAV/1635-1864   1772 FTIVWVIFFLVTCNPAAGILAGVFLVL--LWILGRFT------ 1806
RtEiAV/1521-1749     1657 LTMLWVHFFLVVCNPAAGILSVVLVIM--FWTLGRFT------ 1691
RtMcAV/1522-1751     1659 LTILWVQFFLVVCNAPAAMLAFVLLVC--LWVLGKYT------ 1693
PRRSV-2/1580-1809    1717 LTILWLVFFLISVNMPSGILAMVLLVS--LWLLGRYT------ 1751
PRRSV-1/1464-1693    1601 LTMLWVHSFLVFCLPAAGILSLGITGL--LWAIGRFT------ 1635
RtClanAV/1410-1639   1547 ITIVWSCAFLVFVNPPAGAVSITLLIA--AWLLGRAT------ 1581
LDV/1288-1512        1420 VTLVWVQFFLLAVNFWAGVAVAVILIS--SWFLARAT------ 1454
```

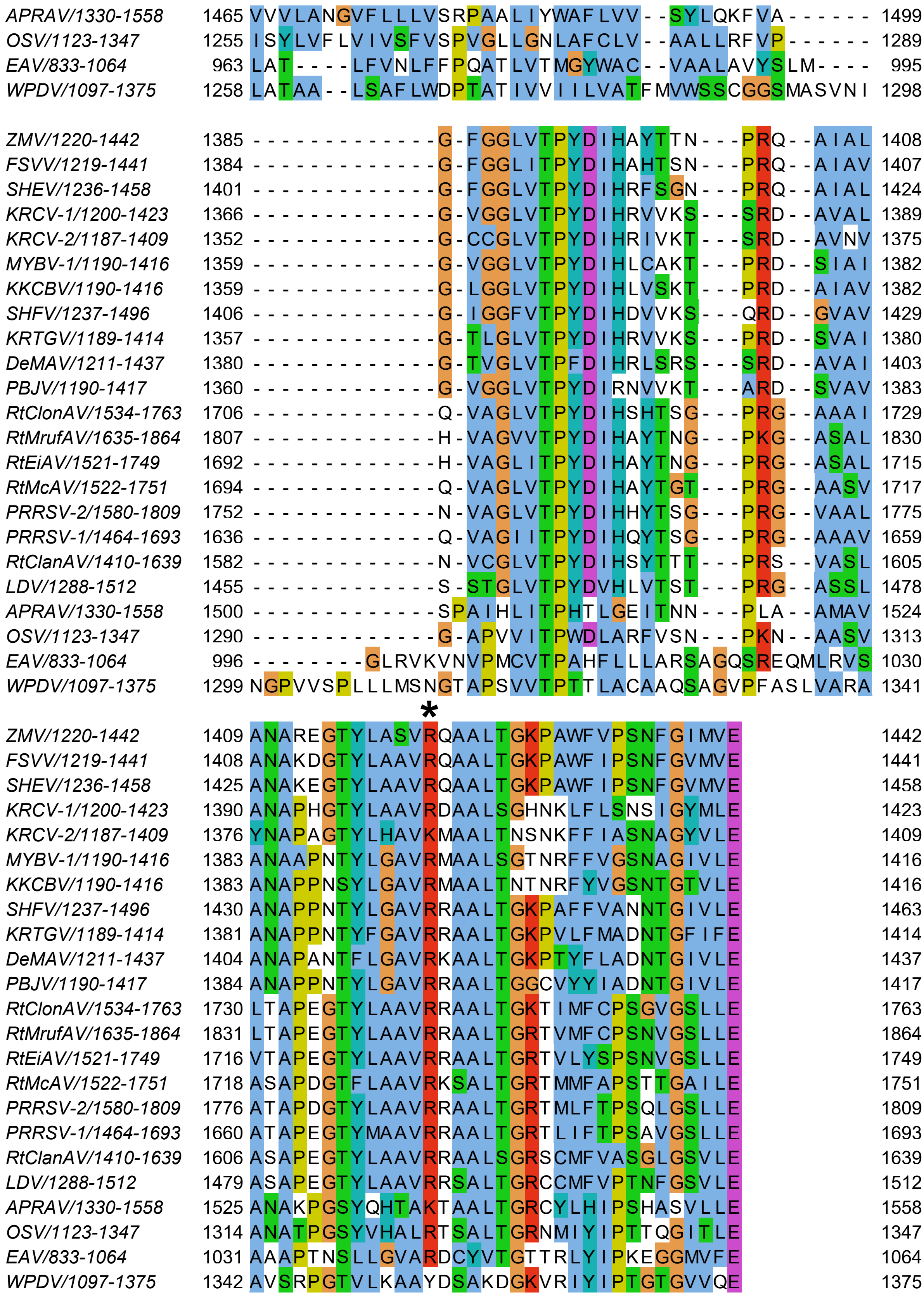

Supplementary Figure 4: Alignment of nsp3 across all currently known arterivirus species.

The first residue in the alignment corresponds to the nsp2/3 cleavage site and the last residue to the nsp3/4 cleavage site. Residues were colored according to the Clustal coloring scheme, with conserved positively-charged residues selected for mutagenesis studies indicated by black asterisks.

Supplementary Figure 5

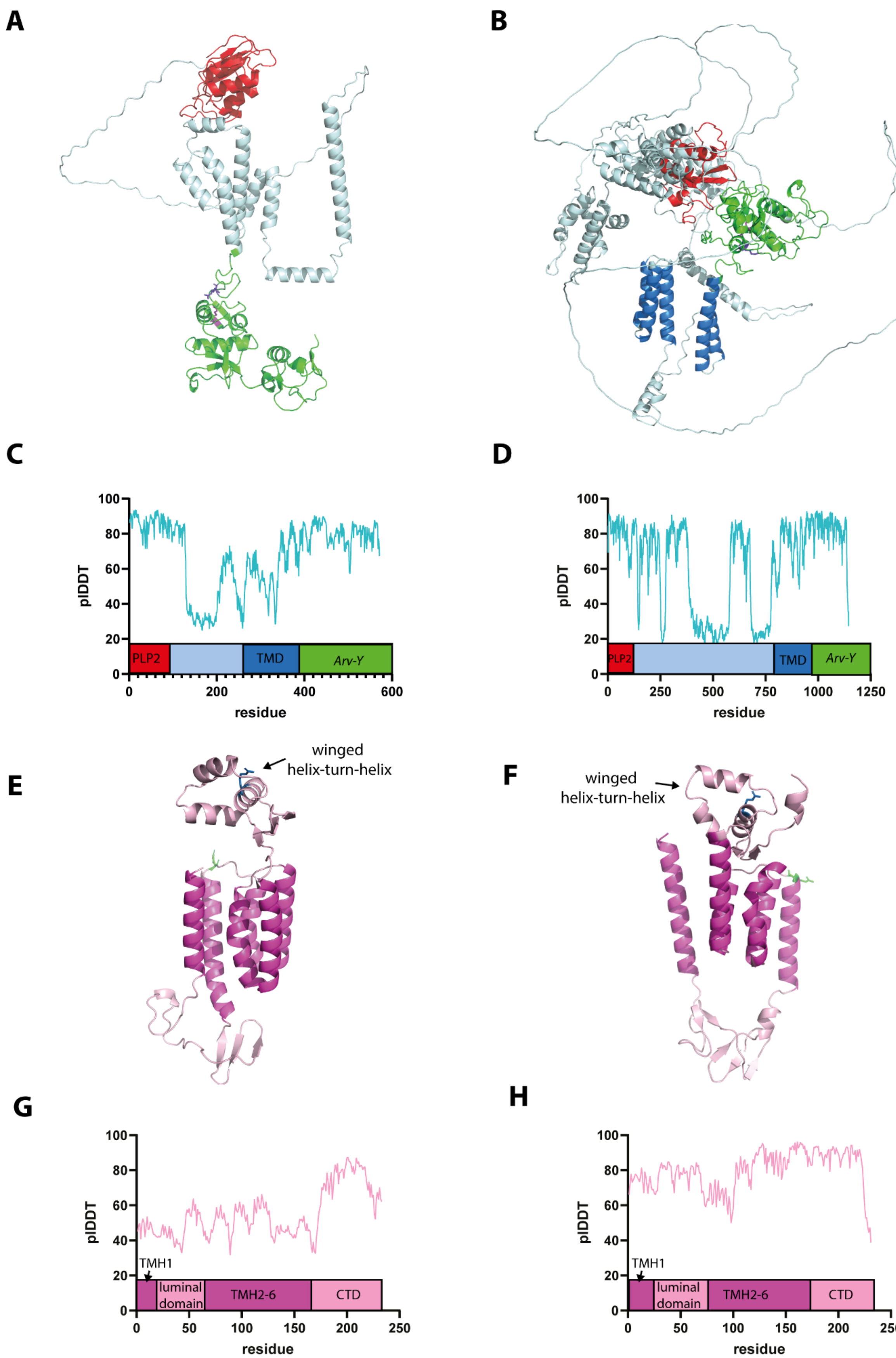

Supplementary Figure 5: Alphafold predictions of EAV and PRRSV nsp2 and nsp3 monomers.

AlphaFold predictions for EAV nsp2 (A) and PRRSV2 nsp2 (B), with PLP2 depicted in red, the previously predicted TM domains in dark blue, and the C-terminal domain in green. The pLDDT plots for EAV nsp2 (C) and PRRSV2 nsp2 (D) with the relevant domains indicated in their respective colors below the graph. Nsp3 of EAV (E) and PRRSV 2(F) are depicted in light pink, with the predicted transmembrane helices indicated in magenta. The pLDDT plots for EAV nsp3 (G) and PRRSV2 nsp3 (H) with the relevant domains indicated in their respective colors below the graph.

Supplementary Figure 6

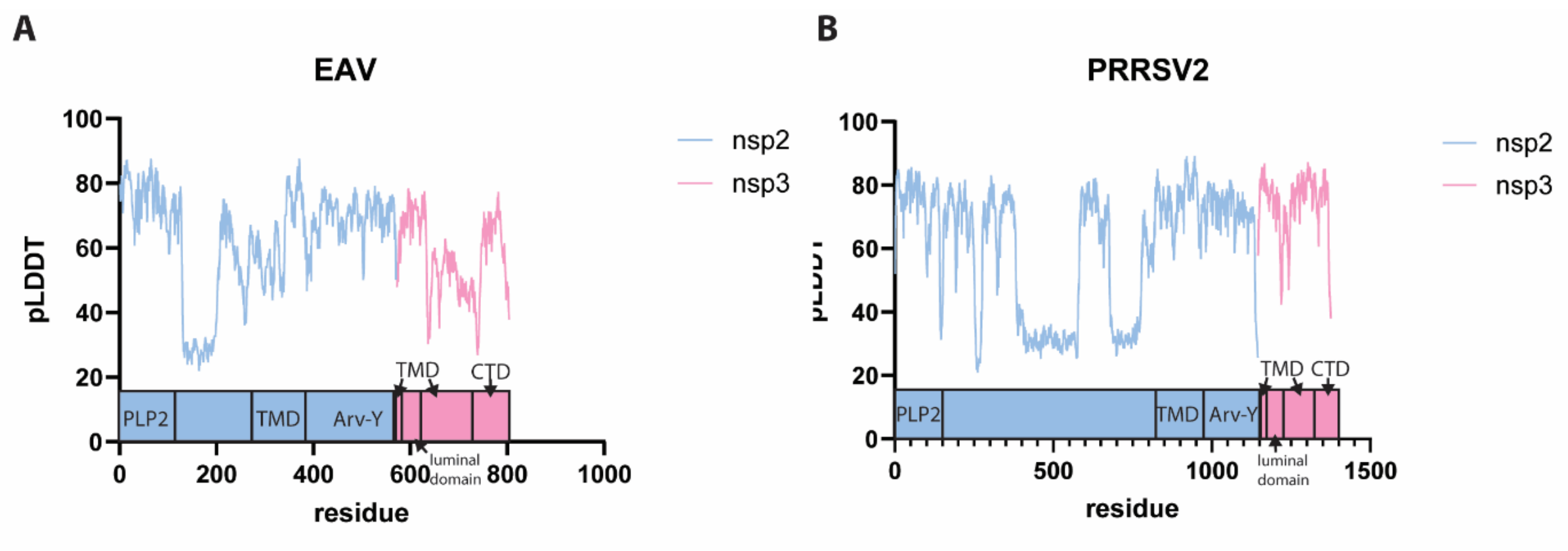

Supplementary Figure 6: pLDDT plots of the nsp2-3 heterodimers predicted by AlphaFold.

The plDDT plots of EAV nsp2-3 (A) and PRRSV2 nsp2-3 (B) with nsp2 depicted in light blue and nsp3 depicted in pink.

Supplementary Figure 7

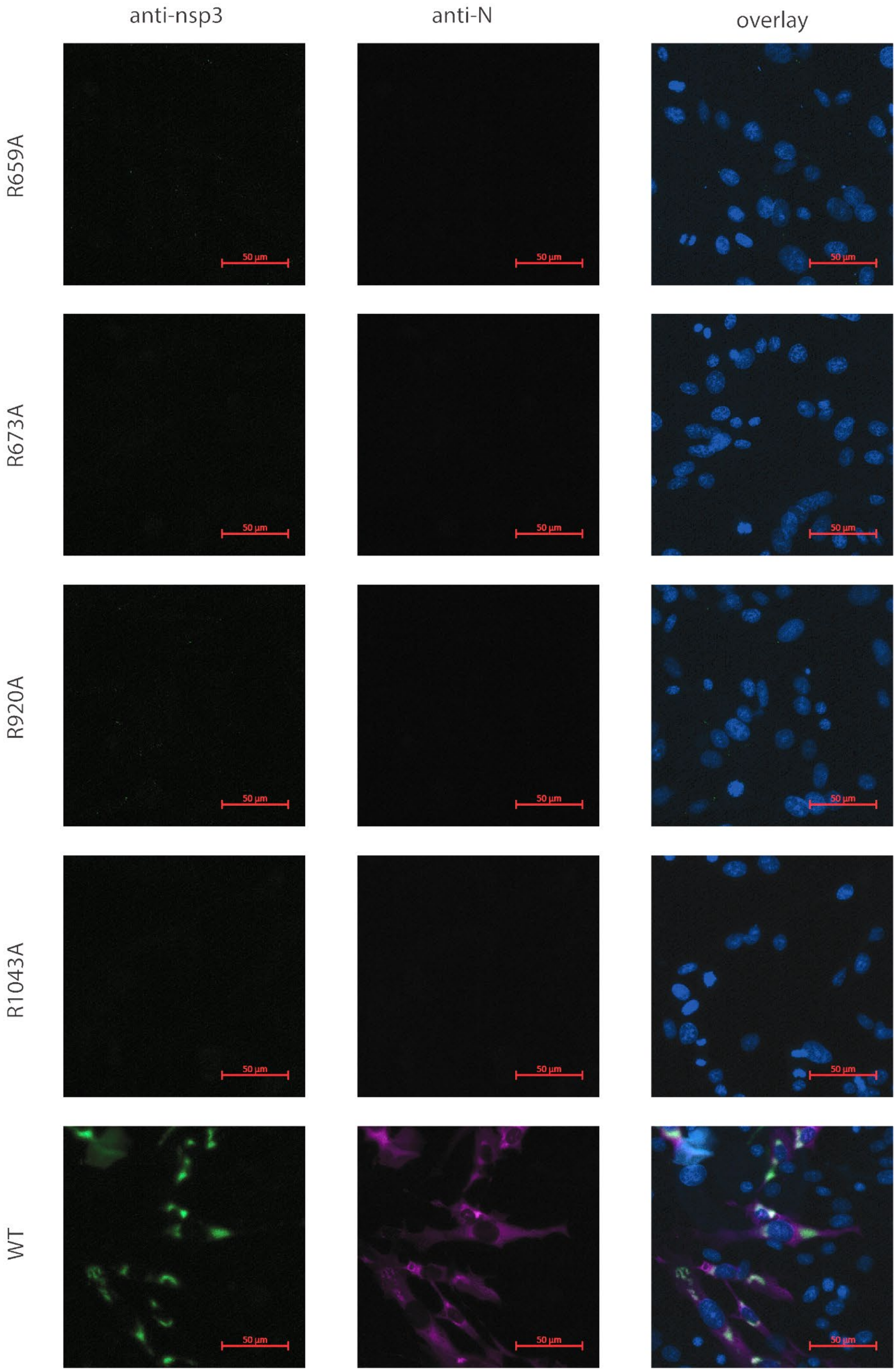

Supplementary Figure 7. Immunofluorescence analysis of Arg-to-Ala mutants reveals lack of detectable expression of viral non-structural or structural proteins.

BHK-21 cells were fixed 16 h post-transfection of the indicated mutant viruses, with recombinant wild-type (WT) virus included as a positive control. Cells were subsequently stained for the nonstructural protein nsp3 (green) and the structural nucleocapsid protein N (magenta). Images were acquired using a 20× objective and equal exposure times. Panels show magnified views of the bottom-right region of each image. Brightness and contrast were adjusted uniformly across the entire figure. While the WT control exhibited clear staining for both viral markers, nsp3 or N protein signals could not be detected for any of the non-viable mutants, suggesting they are negative for both genome replication and subgenomic mRNA synthesis.
